# A Synergistic Dual‐Antibody Cocktail Targeting α‐Toxin Protects Against Invasive *Staphylococcus aureus* Infection by Neutralizing Virulence and Enhancing Host Defense

**DOI:** 10.1002/mco2.70830

**Published:** 2026-07-05

**Authors:** Liqun Zhao, Zhen Song, Hongyin Fan, Lei Wang, Yun Yang, Haiming Jing, Xin Xia, Yu Wang, Leilei Feng, Sheng Wang, Zhifu Chen, Qiang Gou, Yue Yuan, Jinyong Zhang, Quanming Zou, Hao Zeng

**Affiliations:** ^1^ Department of Microbiology and Biochemical Pharmacy National Engineering Research Center of Immunological Products College of Pharmacy and Laboratory Medicine Third Military Medical University Chongqing China; ^2^ Clinical Laboratory Department Army 954th Hospital General Hospital of Tibet Military Region Tibet China; ^3^ CAS Key Laboratory of Infection and Immunity National Laboratory of Macromolecules Institute of Biophysics Chinese Academy of Sciences Beijing China; ^4^ Research and Development Department Chengdu Olymvax Biotechnology Co., Ltd Chengdu China; ^5^ State Key Laboratory of Trauma and Chemical Poisoning Third Military Medical University Chongqing China

**Keywords:** antibiotic synergy, host defense, invasive infection, monoclonal antibodies, *Staphylococcus aureus*, α‐toxin (α‐hemolysin)

## Abstract

*Staphylococcus aureus* is a leading cause of severe invasive infections and is associated with high mortality. The pore‐forming α‐toxin (Hla) is a key virulence determinant that drives disease severity. We identified two high‐affinity anti‐Hla monoclonal antibodies, Hm0399 and Hm0411, derived from the sera of volunteers vaccinated with the *S. aureus* vaccine rFSAV. Mechanistic analyses—including toxin neutralization assays, structure solution, and effector‐function characterization—revealed that although both antibodies recognize overlapping epitopes on Hla, they confer protection through distinct and complementary mechanisms. Hm0411 potently neutralizes Hla toxicity by blocking receptor engagement through a defined salt‐bridge network, whereas Hm0399 engages a hydrogen‐bond interface that enhances Fc‐mediated effector functions. A dual‐antibody cocktail comprising Hm0399 and Hm0411 (Hm3‐4) demonstrated robust therapeutic efficacy in the USA300 *S. aureus* sepsis model and pneumonia model. Notably, protection was not compromised by pre‐existing immune imprinting. Transcriptomic profiling indicated enhanced Fcγ receptor–mediated phagocytosis and suppression of pro‐inflammatory Ca^2+^ signaling pathways. In addition, combining Hm3‐4 with ultra‐low doses of vancomycin or linezolid synergistically improved therapeutic outcomes in experimental sepsis. Collectively, these findings establish a dual‐antibody strategy targeting Hla that integrates virulence neutralization with augmentation of host immune defense, providing a framework for next‐generation immunotherapies against invasive *S. aureus* infection.

## Graphical abstract

Two Hla‐targeting monoclonal antibodies (Hm0399, Hm0411) were isolated from rFSAV‐vaccinated volunteers. Their cocktail (Hm3‐4) showed synergistic anti‐*S. aureus* activity. Hm0411 blocks Hla‐receptor binding via salt bridges; Hm0399 potentiates Fc effector functions through hydrogen bonds. Hm3‐4 also synergizes with ultra‐low‐dose vancomycin/linezolid, substantially boosting therapeutic efficacy.

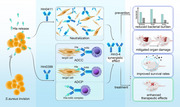

## 1 Introduction

Antimicrobial resistance (AMR) represents a critical global health crisis that compromises the ability to treat infections and safely deliver modern medical care. This escalating threat has been associated with substantial increases in global morbidity and mortality [1], with children being particularly vulnerable. A persistent shortage of effective antimicrobial agents, driven by limited innovation and discovery, further exacerbates this challenge [2, 3, 4, 5]. Consequently, the development of alternative therapeutic strategies—including vaccines, monoclonal antibodies (mAbs), and host‐directed therapies—is urgently needed to combat resistant bacterial infections [6, 7, 8].

Here, we focus on preventive and therapeutic strategies targeting multidrug‐resistant *Staphylococcus aureus*. As an invasive human pathogen, *S. aureus* causes severe hospital‐ and community‐acquired infections, including sepsis and pneumonia, and is associated with high mortality rates [9, 10]. Methicillin‐resistant *S. aureus* (MRSA) is estimated to account for approximately 323,000 hospitalizations, over 10,000 deaths, and $1.7 billion in direct annual economic losses in the United States alone [11]. Humans are the natural hosts of *S. aureus*; studies indicate that more than 50% of individuals are colonized or infected within 2 months of birth, and exposure often persists throughout life [12, 13, 14, 15, 16, 17]. Although certain antibiotics remain effective in clearing *S. aureus*, posttreatment microvascular thrombosis may persist or even worsen, potentially due to the release of bacterial proteins or toxins into the bloodstream during bacterial lysis [18, 19]. Platelets play a key role in anti‐infective defense, as demonstrated by their rapid recruitment to encapsulate *S. aureus* captured by Kupffer cells in hepatic sinusoids [20, 21], thereby limiting systemic dissemination [22]. This early innate immune response eliminates most bacteria; however, approximately 4–8 h after infection [23], a subset of intracellular *S. aureus* within macrophages secretes sufficient α‐toxin (α‐hemolysin; Hla) to induce host‐cell lysis and promote bacterial dissemination [23, 24].

Studies across multiple infection models, including brain abscess, sepsis, pneumonia, and peritonitis, have shown that elevated Hla levels exacerbate disease severity and reduce survival rates [25, 26, 27, 28]. Community‐associated MRSA (CA‐MRSA) strains express higher levels of Hla compared with those of hospital‐associated MRSA (HA‐MRSA) strains, suggesting that enhanced toxin production contributes to the susceptibility of otherwise healthy individuals to invasive *S. aureus* infection [29]. As a central virulence factor that enables immune evasion, Hla binds the metalloprotease ADAM10 (a disintegrin and metalloproteinase domain‐containing protein 10) and assembles into a homomeric heptameric pore, resulting in lysis of epithelial cells, endothelial cells, and platelets [30, 31]. At sublytic concentrations, Hla induces platelet aggregation and hepatic deposition, leading to extensive liver injury and activation of sepsis‐associated inflammatory cascades [24]. Moreover, Hla disrupts epithelial–endothelial barrier integrity by activating ADAM10‐mediated E‐cadherin cleavage, contributing to epithelial damage characteristic of *S. aureus* pneumonia.

In previous work, we developed a multicomponent *S. aureus* vaccine (rFSAV) containing five antigens, including Hla, which is currently undergoing Phase III clinical evaluation (ChiCTR2200062998). Using single‐B‐cell sequencing of peripheral blood mononuclear cells (PBMCs) from rFSAV‐vaccinated volunteers, we identified and characterized two high‐affinity anti‐Hla mAbs, Hm0399 and Hm0411. Following systematic functional evaluation in vitro and in vivo, we developed a dual‐antibody cocktail designed to achieve both toxin neutralization and enhancement of host immune effector activity. Given that CA‐MRSA strains typically express high levels of Hla, we utilized the representative strain USA300 (FPR3757) to assess the efficacy of the dual‐antibody cocktail. In multiple lethal models of USA300‐induced invasive infection, this synergistic strategy demonstrated robust protection against diverse drug‐resistant clinical isolates. Notably, the strategy also overcame immune imprinting interference and prevented reinfection. Mechanistically, the synergy arises from distinct epitope topologies that govern complementary antigen–antibody interactions. These findings provide a framework for the development of anti‐toxin antibody‐based interventions for invasive *S. aureus* infection.

## 2 Results

### 2.1 Generation and Characterization of Anti‐Hla Monoclonal Antibodies

**FIGURE 1:**
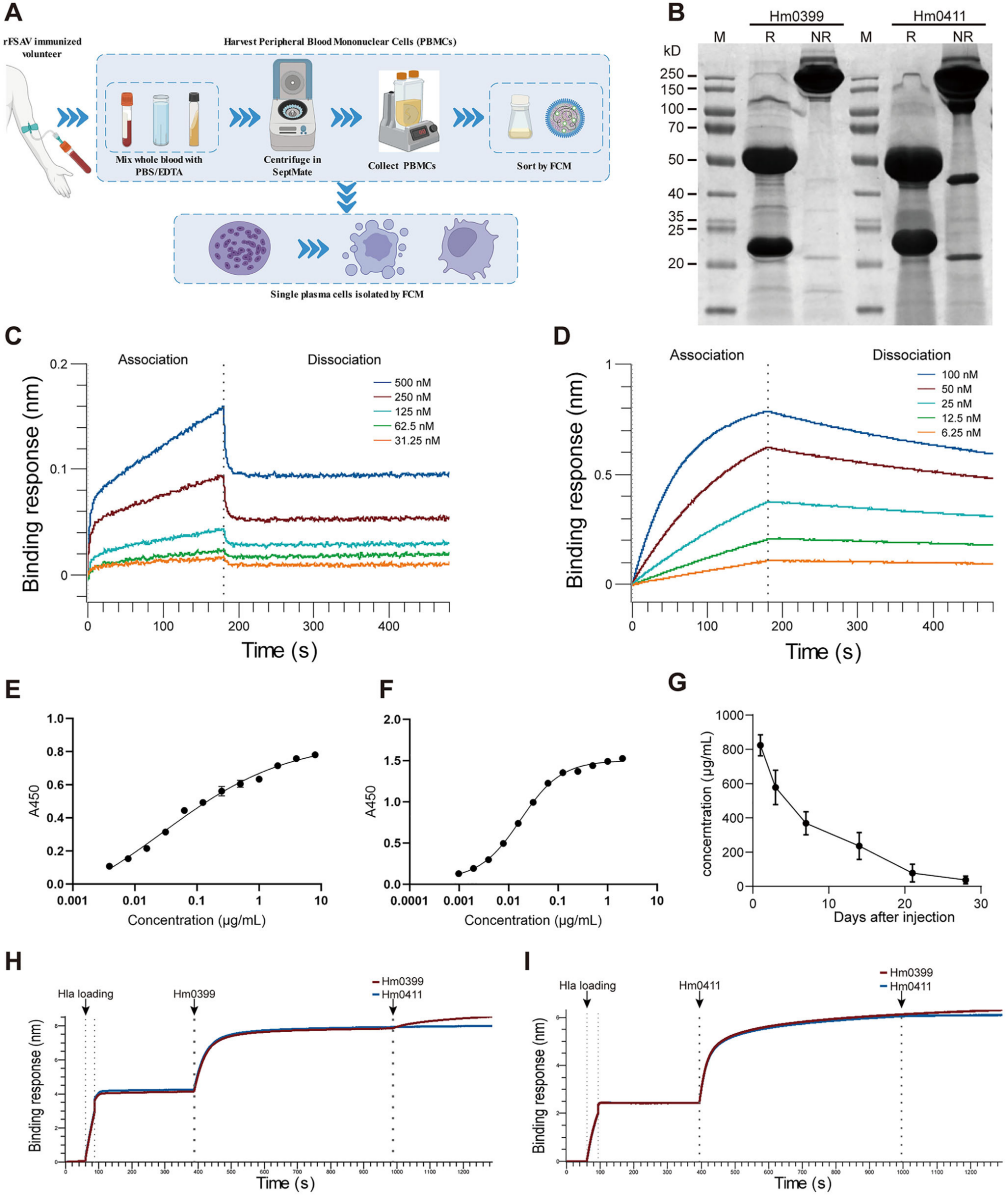
Isolation and characterization of Hm0399 and Hm0411. (A) The mAbs were derived from the PBMCs of clinical volunteers vaccinated with a *S. aureus* vaccine. The overall procedure is shown. (B) The mAbs were purified and verified by SDS‐PAGE. R, reductive electrophoresis; NR, nonreductive electrophoresis. (C, D) The affinity of Hm0399 (C) and Hm0411 (D) was detected by BLI. (E, F) The binding activity of Hm0399 (E) and Hm0411 (F) was detected by ELISA. (G) Pharmacokinetic surveillance of Hm3‐4 in mice was performed by ELISA. (**H, I**) The competitive assay of Hm0399 (**H**) and Hm0411 (**I**) was performed using BLI. Data are presented as mean ± SD and analyzed using GraphPad Prism software (v.10.1.2). Data processing and fitting were performed using variable slope (four parameters) in (E–G).

From the sera of clinical volunteers immunized with the *S. aureus* vaccine (rFSAV), we isolated two anti‐Hla mAbs, Hm0399 and Hm0411 (Figure 1A). Recombinant expression of the corresponding immunoglobulin gene sequences (Table S1) in HEK293F suspension cells yielded antibodies with >95% purity (Figure 1B).

Both mAbs exhibited high binding affinities for Hla. Biolayer interferometry (BLI) determined dissociation constants (K_D_) of 5.34 nM for Hm0399 and 5.50 nM for Hm0411 (Figure 1C,D). Notably, Hm0399 displayed a relatively rapid dissociation rate (K_off_ = 1.15 × 10^−3^ **s^−1^**). Enzyme‐linked immunosorbent assay (ELISA)‐based analysis revealed half‐maximal effective concentrations (EC_50_) of 0.028 µg/mL for Hm0399 and 0.017 µg/mL for Hm0411 (Figure 1E,F).

Pharmacokinetic analysis in mice showed that both antibodies maintained sustained serum levels for up to 15 days after administration (Figure 1G). Epitope competition assays demonstrated that, following immobilization of Hla bound to Hm0399 (or Hm0411), subsequent addition of Hm0411 (or Hm0399) produced only minimal changes in detection signal. These findings indicate that the two antibodies recognize comparable epitopes on Hla (Figure 1H,I).

### 2.2 Evaluation of Toxin‐Neutralizing Activity of Hm0399 and Hm0411

**FIGURE 2:**
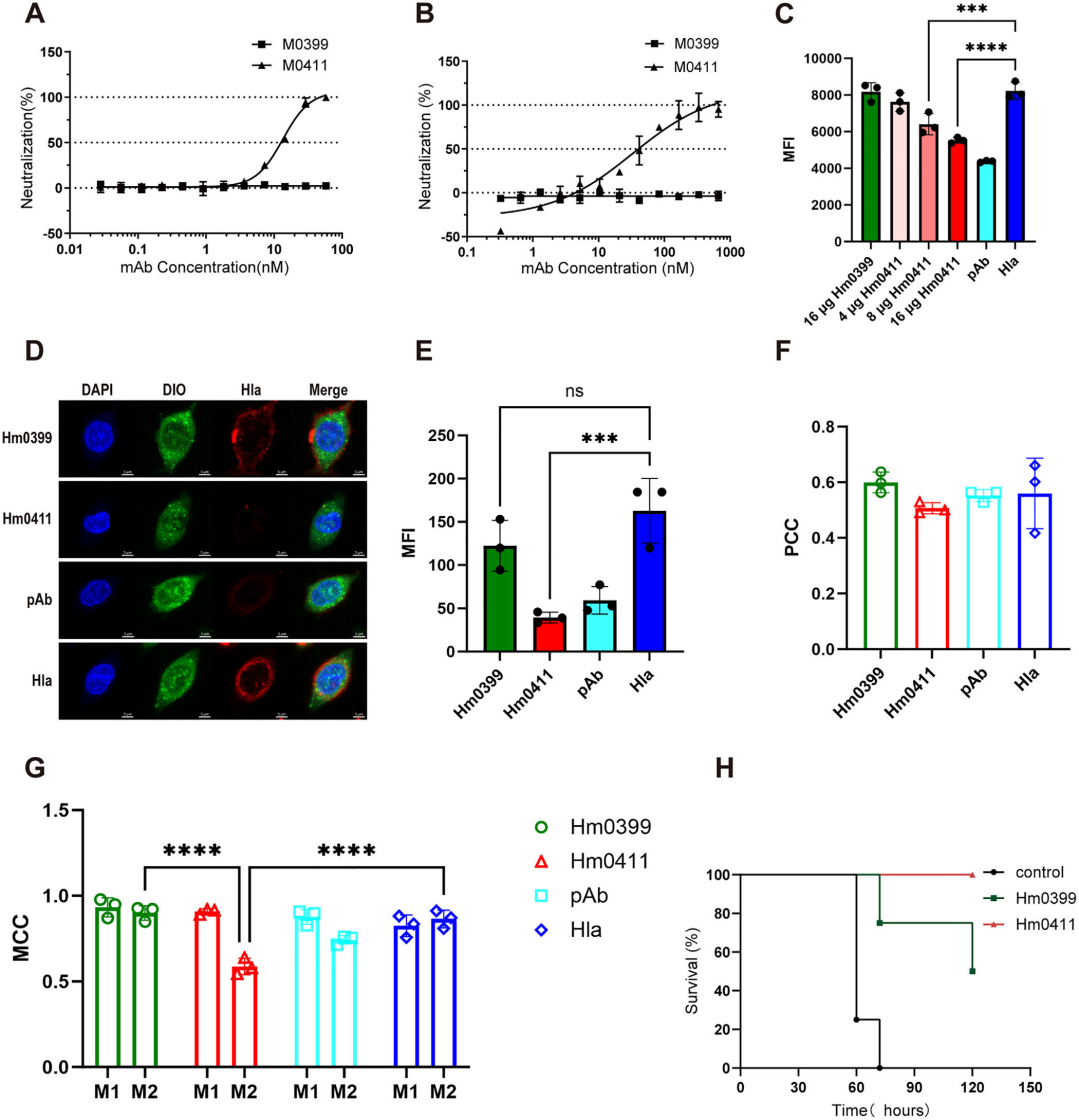
The neutralization activity of Hm0399 and Hm0411. (A, B) Hla (25 ng) was incubated with a concentration series of Hm0399 and Hm0411, and the mixture was added to rabbit RBCs (A) and A549 cells (B); cell lysis was then detected. (C–E) Cy5‐labeled Hla was incubated with the mAbs, and the mixture was added to A549 cells. The blocking of Hla binding to A549 cells was detected by flow cytometry (C) (*n* = 3) and laser scanning confocal microscopy. DIO, 1,1'‐Dioctadecyl‐3,3,3',3'‐tetramethylindocarbocyanine perchlorate, representing membrane (D). Mean fluorescence intensity (MFI) was calculated using Image J software (E). (F, G) The Pearson's correlation coefficient (PCC) (F) and Manders’ colocalization coefficient (MCC) (G) of Cy5‐Hla and the membrane. Three independent images were collected, and the MFI of Cy5 was calculated. (H) A lethal dose of the Hla toxin was intravenously administered to BALB/c mice, and the mAbs were intravenously injected at 30 min post‐toxin administration. Survival was then measured. *n* = 5 mice per treatment group; data are presented as mean ± SD and analyzed using GraphPad Prism software (v.10.1.2). Data processing and fitting were performed using variable slope (four parameters) in (A, B). *p*‐values were determined using ordinary one‐way ANOVA with Dunnett's multiple comparison in (C), (E), and (F); *p*‐values were determined using two‐way ANOVA with Šidák correction in (G); *p*‐values were determined using simple survival analysis (Kaplan–Meier) in (H). ns, not significant; *p < 0.05; **p < 0.01; ***p < 0.001; ****p < 0.0001.

The neutralizing activities of Hm0399 and Hm0411 against Hla were assessed using rabbit red blood cells (RBCs) and A549 human lung epithelial cells (RRID: CVCL_C8WF). Hm0411 demonstrated potent, dose‐dependent inhibition of Hla‐mediated hemolysis (EC_50_ = 13.47 nM), whereas Hm0399 exhibited no detectable neutralizing activity. Similarly, in cytotoxicity assays, Hm0411 effectively inhibited Hla‐induced A549 cell death (EC_50_ = 33.13 nM), in contrast to the lack of inhibition observed with Hm0399 (Figure 2A,B).

Flow cytometric analysis confirmed a substantial decrease in Cy5‐Hla binding to A549 cells following Hm0411 treatment (Figure 2C). Laser‐scanning confocal microscopy (LSCM) showed that Hm0411 markedly reduced the binding of Cy5‐labeled Hla to A549 cells, as reflected by significantly lower mean fluorescence intensity compared with controls (Figure 2D,E). Pearson's correlation coefficient (PCC) was calculated and presented in Figure 2F, indicating moderate co‐localization between the two channels. Given that the Manders’ colocalization coefficient (MCC) more accurately quantifies the actual proportion of overlapping signals, we further computed the M1 (fraction of Channel 1 overlapping with Channel 2) and M2(fraction of Channel 2 overlapping with Channel 1) values. As shown in Figure 2G, M2 of the Hm0399 group was significantly larger than the Hm0411 group (*p* < 0.0001). And M2 of the Hm0411 group was significantly smaller than the Hla group (*p* < 0.0001).

To extend these observations in vivo, we established a murine model of lethal Hla intoxication. BALB/c mice received an intravenous (i.v.) lethal dose of Hla (2 µg per mouse). Thirty minutes postchallenge, mice were therapeutically administered Hm0411 (4 µg per mouse) or Hm0399 (4 µg per mouse) intravenously and monitored for 120 h. Hm0411 treatment conferred complete protection (100% survival) with no significant pathological findings. In contrast, Hm0399‐treated mice showed 40% survival (Figure 2H). These in vivo outcomes were consistent with the in vitro neutralization results.

Together, these findings indicate that Hm0411 potently neutralizes Hla‐mediated hemolysis and cytotoxicity in vitro and protects against lethal Hla intoxication in vivo, likely by blocking Hla binding to host‐cell receptors. In contrast, Hm0399 showed minimal direct neutralizing activity against Hla.

### 2.3 Cryo‐EM Reveals the Structural Basis of Antibody–Hla Interactions

Previous experiments demonstrated that Hm0411 and Hm0399 bind monomeric Hla and recognize comparable epitopes; however, only Hm0411 exhibited potent neutralizing and receptor‐blocking activity, whereas Hm0399 lacked measurable neutralizing efficacy. Because Hla pathogenicity is primarily mediated by the formation of a heptameric pore, structural analyses based solely on monomeric Hla may not fully recapitulate the physiological context. We therefore asked whether the epitopes recognized by these antibodies remain accessible following heptamer assembly and hypothesized that their functional divergence reflects differences in spatial binding modes to the Hla heptamer, including binding angle, epitope accessibility, and steric constraints.

**FIGURE 3:**
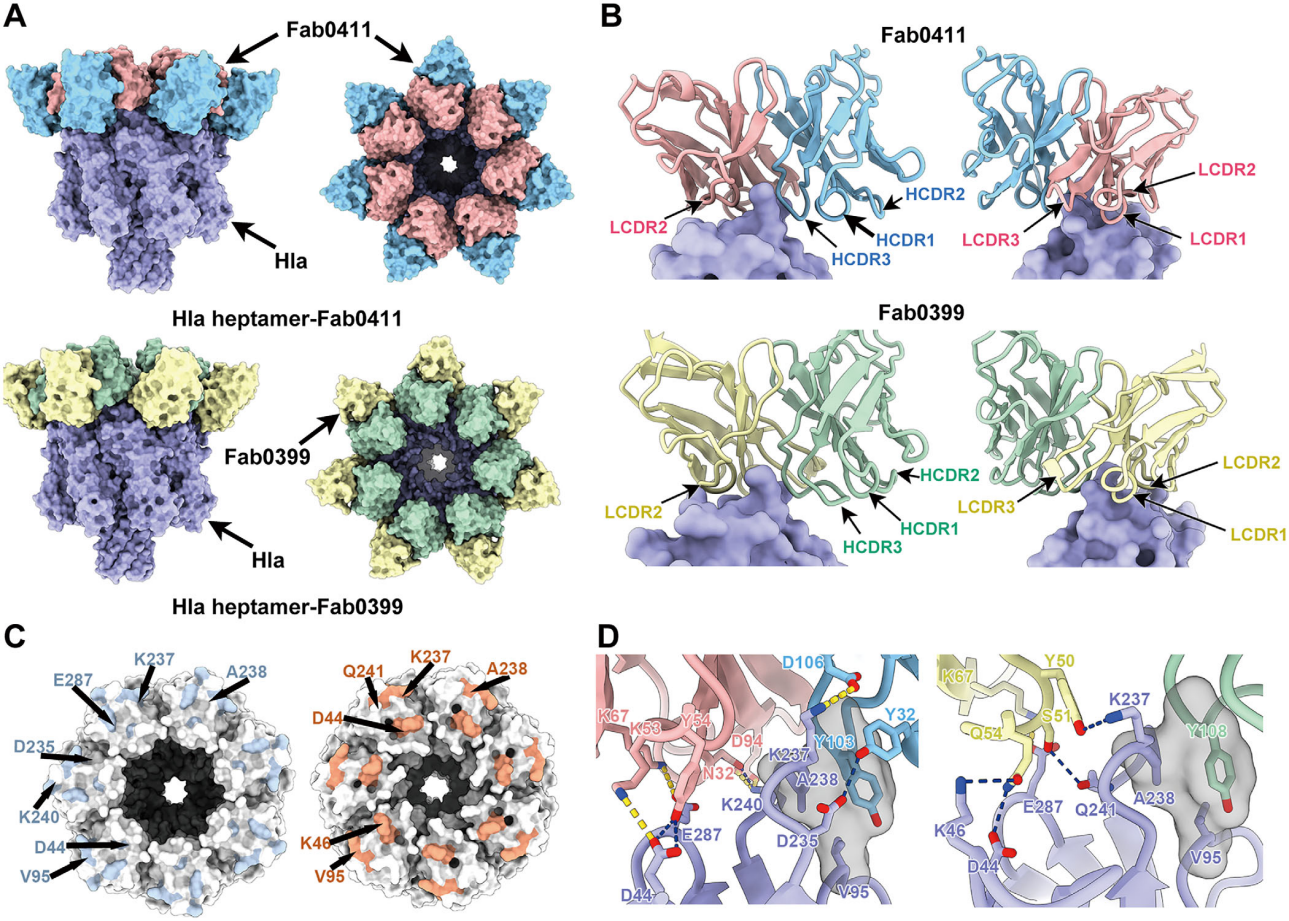
Structural and molecular basis of Fab0411 and Fab0399 binding to Hla heptamer. (A) Overall structures of Fab0411/Hla and Fab0399/Hla complexes. Complexes are shown in surface representation, with Fab0411 heavy chain colored in blue and light chain in pink; Fab0399 heavy chain is shown in green and light chain in yellow; Hla is shown in purple. (B) Contribution of the heavy and light chains of Fab0411 and Fab0399 to Hla binding. Hla is represented on the surface. Fabs are shown using a cartoon style. The colors displayed are the same as in previous panel A. (C) Epitopes of Fab0411 (light blue) and Fab0399 (light salmon) on Hla heptamer. (D) Interactions between Fab0411 (left) and Fab0399 (right) with Hla. Contact residues are shown as sticks and are labeled; hydrogen bonds are shown as thin blue‐dotted lines, salt bridges are shown as thick, yellow‐dotted lines. Hydrophobic patches are shown in gray surface representation. The color scheme is the same as that in panel A.

To test this hypothesis, we determined cryo‐electron microscopy (cryo‐EM) structures of the Hm0411–Hla and Hm0399–Hla heptamer complexes at resolutions of 2.79 and 3.09 Å, respectively, using single‐particle analysis (Figure S2). The high‐resolution density maps enabled precise delineation of conformational epitopes at the Hla–Fab interface and identification of key contacts within complementarity‐determining regions (CDRs) (Figure S3). Both Fab‐Hm0411 (Fab0411) and Fab‐Hm0399 (Fab0399) bound the cap domain of the Hla heptamer (Figure 3A), with interactions predominantly mediated by the light chains.

Detailed structural analysis showed that Hla was positioned within a crevice formed by the light‐chain CDRs (LCDR1‐3) and heavy‐chain CDR1 and CDR3 (HCDR1 and HCDR3) of the Fab fragments (Figure 3B). The buried surface area was larger for the Fab0411–Hla interface (665 Å^2^) than for the Fab0399–Hla interface (571 Å^2^), with light chains contributing approximately 60% of the total buried surface area in both complexes.) of the Fab fragments.

Epitope mapping revealed that Fab0411 and Fab0399 recognize comparable epitopes on Hla, sharing key residues D44, V95, K237, A238, and E287. Fab0411 uniquely contacted D235, whereas Fab0399 additionally engaged K46 and Q241 (Figure 3C). Importantly, Fab0411 formed a more extensive interaction network with Hla. Three hydrogen bonds were observed between Hla residues D44 and K240 and Fab0411 light‐chain residues Y54, Y32, and N32, and one additional hydrogen bond formed between Hla D235 and Fab0411 heavy‐chain residue Y32 (Figure 3D; Tables S2 and S3). Notably, Fab0411 also formed five salt bridges with Hla, involving Hla residues D44, K240, and E287 and Fab0411 residues K53, D94, and K67 in the light chain and D106 in the heavy chain. Because salt bridges represent among the strongest noncovalent stabilizing interactions, these contacts likely contribute substantially to the stability of the Hm0411–Hla complex. In addition, a hydrophobic pocket formed by Hla residues V95 and A238, together with Fab0411 heavy‐chain residue Y103, further strengthened binding. By comparison, Fab0399 formed fewer stabilizing interactions with Hla. Only four hydrogen bonds were observed between Hla residues D44, K46, R236, and K237 and Fab0399 light‐chain residues Q54 and Y50. A single salt bridge was detected between Hla residue E287 and Fab0399 heavy‐chain residue K67. This sharply contrasts with the five salt bridges observed in the Fab0411–Hla complex. Fab0399 also formed a hydrophobic pocket similar to that observed in the Fab0411 complex, involving Hla residues V95 and A238 and Fab0399 heavy‐chain residue Y108.

Together, these structural data indicate that although Hm0411 and Hm0399 bind similar epitopes on the Hla heptamer, Hm0411 engages a more extensive and stabilizing interaction network, including multiple salt bridges and hydrophobic contacts. This enhanced interaction network likely underlies the superior neutralizing activity of Hm0411. In contrast, the Hm0399–Hla interface is dominated by hydrogen bond interactions, consistent with the relatively faster dissociation kinetics observed for Hm0399.

### 2.4 Rapid Dissociation Kinetics Enable Fc‐Mediated Effector Functions for Pathogen Clearance

Antibodies confer protection through both Fab‐mediated neutralization and Fc‐mediated effector functions, including antibody‐dependent cellular cytotoxicity (ADCC), antibody‐dependent cellular phagocytosis (ADCP), and complement‐dependent cytotoxicity. Increasing clinical and preclinical evidence highlights the critical contribution of Fc effector activity to optimal protective efficacy [32, 33, 34, 35, 36, 37, 38, 39, 40, 41, 42, 43, 44, 45, 46], particularly in cancer immunotherapy and infectious diseases [44, 47, 48, 49].

Because Hm0399 and Hm0411 recognize overlapping epitopes on Hla, and Hm0411 exhibits stronger neutralizing activity due to more stable antigen binding, we hypothesized that differences in their binding kinetics may differentially influence Fc‐mediated effector functions. To test this hypothesis, we initially exposed A549 cells to Hla and then added Hm0399 or Hm0411 together with effector cells (Figure S4A,B). This approach proved suboptimal: high concentrations of Hla rapidly induced A549 cell death, whereas lower concentrations failed to elicit detectable effector‐cell activation. We therefore established a cellular model based on lentiviral overexpression of Hla and used flow cytometry to evaluate antibody binding to cell‐surface‐expressed Hla. Both Hm0399 and Hm0411 bound stably to HEK293 cells expressing Hla, confirming epitope accessibility and supporting the suitability of this model for evaluating Fc‐mediated effector functions (Figure S4C–F).

**FIGURE 4:**
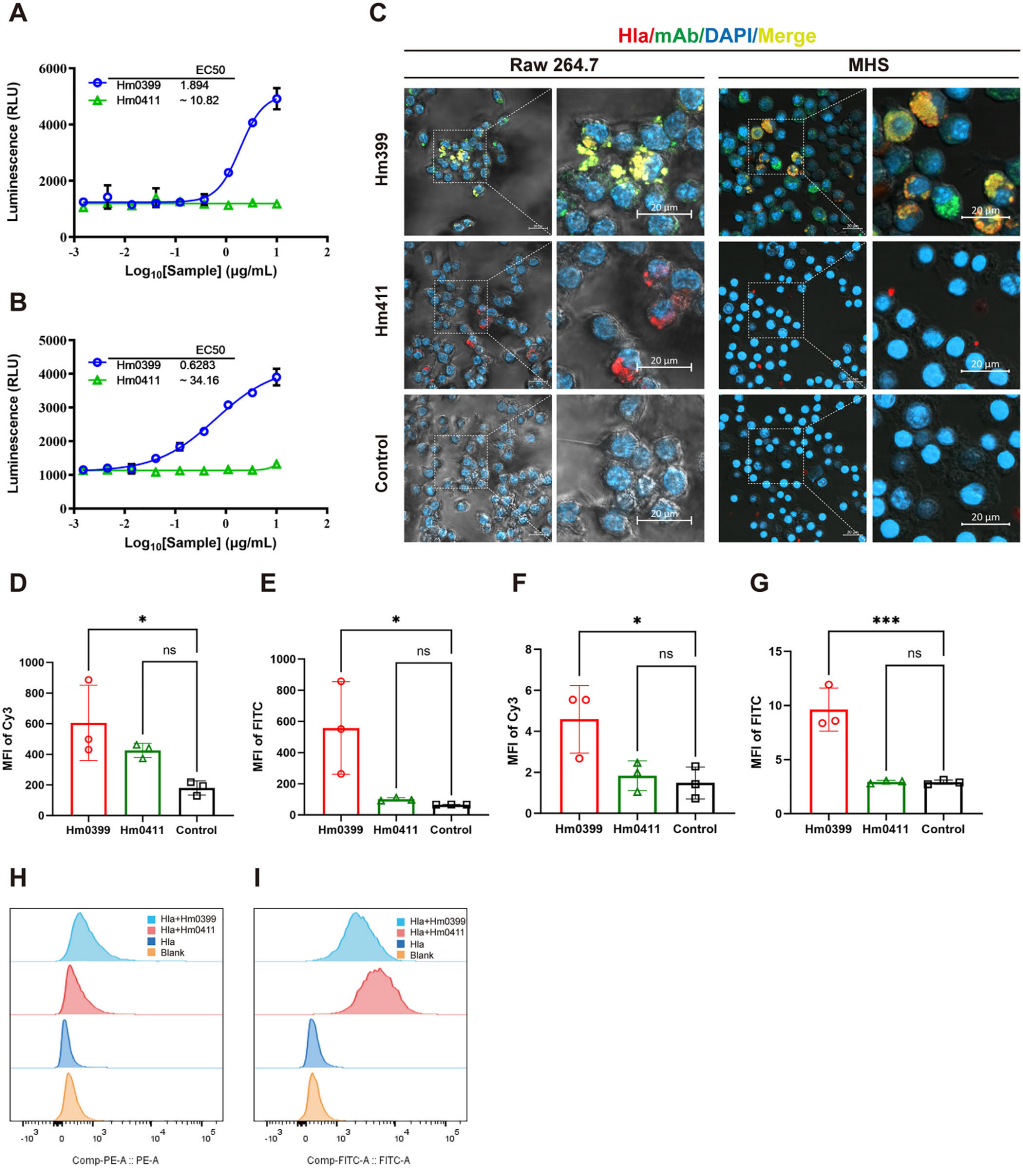
The synergistic effect of the two‐mAb cocktail on toxin clearance and immune restoration via activation of Fc effector functions. (A) Detection of ADCC using GMOne‐Step Luc assay. (B) Detection of ADCP using GMOne‐Step Luc assay. (C–G) Macrophage‐mediated phagocytosis of Cy3‐labeled Hla and FITC‐labeled mAb complex by Raw264.7 and MHS cells was observed using LSCM. Representative images are shown in (C). Three independent images were collected, and the MFI of Cy3 (D) and FITC (E) in Raw264.7 cells was calculated. The MFI of Cy3 (F) and FITC (G) in MHS cells was calculated. (H, I) Macrophage‐mediated phagocytosis of Cy3‐labeled Hla (H) and FITC‐labeled mAb (I) complex by Raw264.7 cells was detected using flow cytometry. Data are presented as mean ± SD and analyzed using GraphPad Prism software (v.10.1.2). Data processing and fitting were performed using variable slope (four parameters) in (A) and (B). *p*‐values were determined by ordinary one‐way ANOVA with Dunnett's multiple comparison in (D–G). ns, not significant; *p < 0.05; **p < 0.01; ***p < 0.001.

Using Jurkat–Fcγ receptor IIIa (FcγRIIIa) and Jurkat–Fcγ receptor IIa (FcγRIIa) reporter cells as effectors for ADCC and ADCP, respectively, Hm0399 demonstrated robust ADCC (EC_50_ = 1.894 µg/mL) and ADCP (EC_50_ = 0.6283 µg/mL) activity. In contrast, Hm0411 showed minimal activation in these assays (Figure 4A,B). These findings support the concept that antibodies with faster dissociation kinetics facilitate more efficient engagement of Fcγ receptors and enhanced recruitment of effector cells, thereby augmenting Fc‐mediated clearance [50]. ADCP contributes not only to the elimination of infected cells but to the clearance of opsonized pathogens and immune complexes, thereby maximizing protective efficacy [51, 52]. To evaluate this function, we generated immune complexes by incubating Hla with either Hm0399 or Hm0411, followed by exposure to phagocytes. Qualitative imaging using LSCM and quantitative flow cytometry showed that both mAbs promoted macrophage‐mediated phagocytosis of immune complexes to a comparable extent (Figure 4C–I). We note that partial dissociation of the more rapidly dissociating Hm0399 during wash steps may limit precise quantification of subtle differences in clearance efficiency. Given the partial epitope overlap between Hm0399 and Hm0411, we also assessed potential interference between the two antibodies. As shown in Figure S4G, Hm0399 did not impair the neutralizing activity of Hm0411. Similarly, Hm0411 did not compromise the effector function of Hm0399 (Figure S4H,I).

Collectively, these findings demonstrate that rapid antibody dissociation kinetics facilitate efficient Fcγ receptor engagement and potent ADCC and ADCP responses, thereby promoting effective pathogen clearance.

### 2.5 Synergistic Dual‐Antibody Cocktail Confers Broad Protection Against *S. aureus* Sepsis

Previous studies established that Hm0411 potently neutralizes Hla by blocking receptor binding but exhibits minimal Fc‐mediated effector activity, whereas Hm0399 activates ADCC and ADCP but lacks direct toxin‐neutralizing activity. Cryo‐EM revealed distinct binding modes despite partial epitope overlap. This functional and structural divergence prompted us to determine, in vivo, which mechanism predominates in the complex context of sepsis and whether combining the two antibodies yields synergistic protection.

**FIGURE 5:**
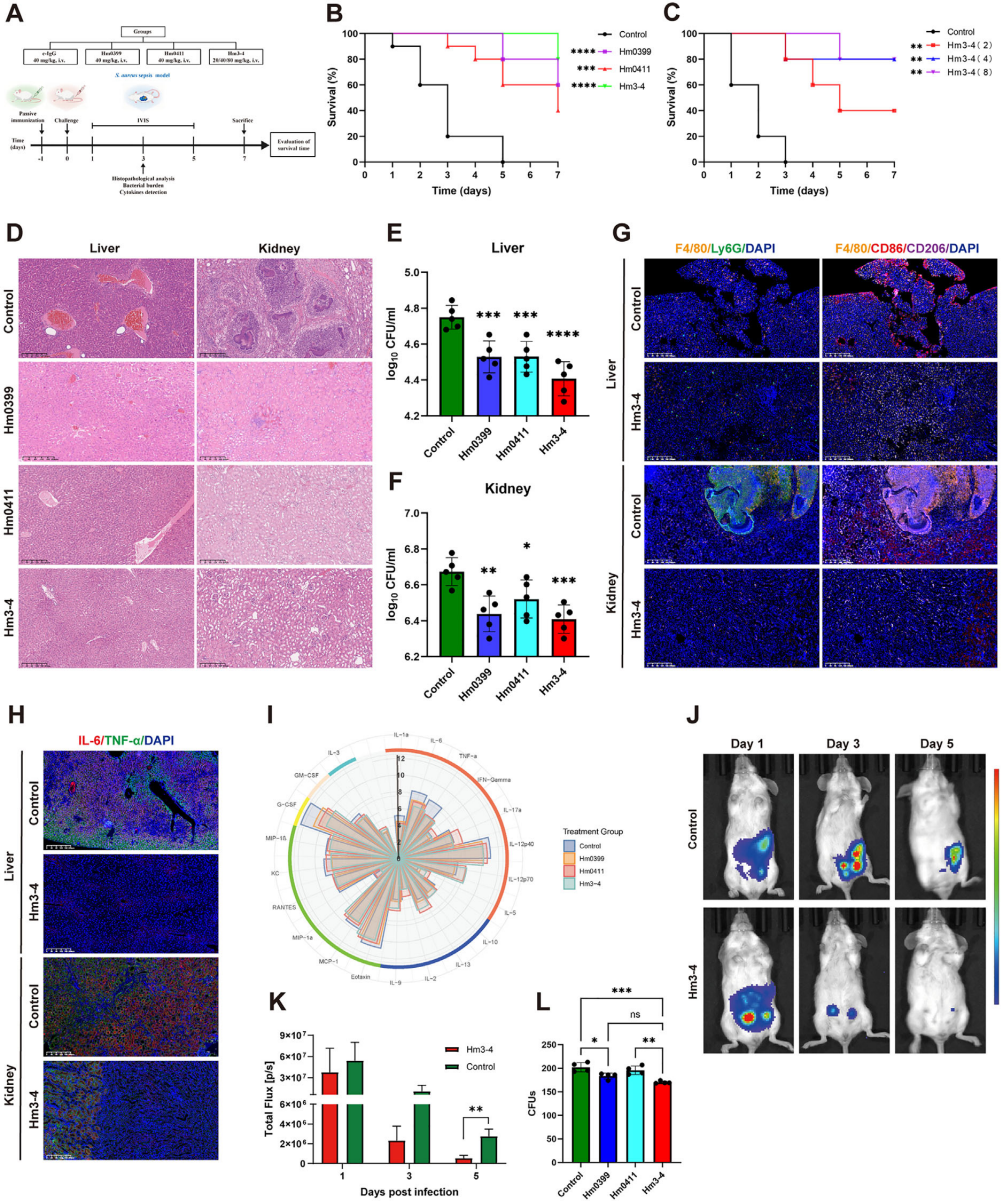
The protective efficacy of the two‐mAb cocktail against *S. aureus* sepsis. (A) In the challenge model, mice were passively immunized with mAbs via i.v. injection 24 h before *S. aureus* challenge. Survival rates were monitored daily for a 7‐day period following the challenge. Liver and kidney tissue of the infection model were subjected to histopathological examination using hematoxylin and eosin (H&E) staining 72 h postinfection. Concurrently, the bacterial load in these organs was quantified by CFU enumeration at the same time point. (B) The protective efficacy of the mAbs in mice subjected to an i.v. lethal‐dose challenge with USA300. (C) The dose‐dependent efficacy of Hm3‐4. *n* = 5 mice. (D) Histopathologic evaluation of the liver and kidney at 72 h postinfection. *n* = 3 mice. (E, F) The bacterial burden in the liver (E) and kidney (F) was determined. *n* = 5 mice. (G, H) Immunofluorescence labeling of cell markers (G), including F4/80, Ly6g, CD86, and CD206, and cytokines (H), including IL‐6 and TNF‐α, in the liver and kidney. *n* = 3 mice. (I) Cytokine analysis in serum was conducted using Luminex. *n* = 3 mice. (J, K) Bacterial clearance was monitored using an IVIS imaging system on days 1 to 5 postinfection. *n* = 3 mice. Representative figures (J) and quantification of bioluminescence signals (K) are shown. (L) The in vitro bactericidal assays. *n* = 4. Data are presented as mean ± SD and analyzed using GraphPad Prism software (v.10.1.2). *p*‐values were determined using simple survival analysis (Kaplan–Meier) in (B, C); *p‐* values were determined using ordinary one‐way ANOVA with Dunnett's multiple comparison in (E), (F), and (L); *p*‐values were determined using unpaired *t*‐test in (K). ns, not significant; *p < 0.05; **p < 0.01; ***p < 0.001; ****P < 0.0001.

Mice received Hm0399 (40 mg/kg), Hm0411 (40 mg/kg), a dual‐antibody cocktail (Hm3‐4; 1:1 ratio of Hm0399:Hm0411 at 20, 40, or 80 mg/kg), or control immunoglobulin G (c‐IgG; 40 mg/kg) intravenously 24 h before lethal challenge with MRSA USA300 (Figure 5A). During the initial 7‐day observation period, all antibody treatments significantly improved survival compared with that of c‐IgG (Figure 5B). Upon extending the observation period to 15 days, Hm3‐4 demonstrated optimal protective efficacy (Figure S5A), with this effect being dose‐dependent. Notably, the 40 mg/kg dose of Hm3‐4 conferred the optimal protective efficacy (Figure 5C). Histopathological analysis at 72 h postinfection showed that Hm3‐4 markedly reduced hepatic and renal injury, including decreased hepatic platelet aggregation and deposition, reduced cellular necrosis, inhibition of renal abscess formation and hyaline thrombi, preservation of renal tubular architecture, and attenuation of inflammatory infiltration in both organs (Figure 5D). Bacterial burdens were significantly lower in Hm3‐4‐treated mice than in c‐IgG controls but were not significantly different from those in the single‐antibody groups (Figure 5E,F), consistent with observations that survival following intravenous *S. aureus* challenge does not always correlate with bacterial load at a single time point.

Based on these results, we selected Hm3‐4 for further mechanistic investigation. Immunofluorescence labeling and cytokine profiling were performed to evaluate host immune responses. CD86, a key co‐stimulatory molecule expressed by monocytes and macrophages and a predictor of early sepsis severity, was significantly reduced in the liver and kidneys of Hm3‐4‐treated mice (Figure 5G). Macrophages represented the predominant immune cell population in infected tissues, and Hm3‐4 treatment promoted polarization toward an M2‐like phenotype (Figure 5G). Expression of the pro‐inflammatory cytokines interleukin (IL)‐6 and tumor necrosis factor‐α (TNF‐α) was significantly decreased in both organs (Figure 5H). Systemic cytokine profiling (23 cytokines; undetectable analytes not shown) confirmed significant downregulation of ten pro‐inflammatory mediators, including TNF‐α and IL‐6, and upregulation of four anti‐inflammatory mediators, including IL‐10 and IL‐13 (Figure 5I). These findings indicate that Hm3‐4 modulates the host immune response, effectively attenuating excessive inflammation and facilitating tissue repair. The core mechanism underlying this effect stems from the synergistic integration of its constituent antibodies, Hm0411 and Hm0399. Hm0411 binds with high affinity to Hla, efficiently neutralizing it. This blockade prevents both Hla‐induced direct macrophage damage and its sustained immune‐stimulatory effects, thereby inhibiting the dysregulated overproduction of pro‐inflammatory cytokines triggered by Hla. Concurrently, Hm0399 leverages potent ADCC and ADCP to accelerate the phagocytic clearance of Hla‐damaged host cells and immune complexes. This clearance process directly: (1) drives macrophage polarization toward an M2‐like anti‐inflammatory/reparative phenotype, interrupting inflammatory amplification; (2) reduces persistent antigen exposure, further suppressing pro‐inflammatory factor production; and (3) synergizes with the induced M2‐like phenotype to enhance the release of reparative cytokines. The functional synergy between Hm0411 and Hm0399 thus constitutes the foundation of Hm3‐4's comprehensive immunomodulatory capacity, underpinning its robust in vivo protective efficacy.

In addition, Hm3‐4 enhanced the host's ability to clear *S. aureus*, as demonstrated through real‐time in vivo monitoring of bacterial clearance in mice (Figure 5J,K) and in vitro bactericidal assays (Figure 5L). To evaluate the broad‐spectrum protective efficacy of Hm3‐4, we selected clinically representative *S. aureus* isolates for testing. These strains encompassed major lineages, including ST59 (CA‐MRSA, CC59), Newman (methicillin‐susceptible *S. aureus* [MSSA], CC88), NCTC8325‐4 (MSSA, CC8), and its isogenic *hla*‐knockout strain (NCTC8325‐4 Δ*hla*). The results demonstrated that Hm3‐4 conferred robust protection against all tested strains. Importantly, its efficacy was unaffected by either the methicillin resistance status (MRSA vs. MSSA) or the genetic background of the strains (Figure S5B–D). Notably, the Δ*hla* strain required at least twofold higher inoculum to induce lethal sepsis. Consistent with the specificity of the cocktail for Hla, Hm3‐4 did not improve survival in mice challenged with the Δ*hla* strain compared with c‐IgG (Figure S5E). This finding confirms the high specificity of Hm3‐4 for its target, Hla, and underscores the pivotal role of Hla in *S. aureus* sepsis pathogenesis.

Considering that humans experience repeated natural exposure to *S. aureus*, distinct from the experimental mouse model, we established a pre‐exposure model based on the study by Tsai et al. [53]. In this model, BALB/c mice received three intraperitoneal (i.p.) injections of USA300 per week for pre‐exposure, followed by intravenous (i.v.) administration of Hm3‐4. Twenty‐four hours later, mice were challenged via i.v. injection of USA300 or i.p. injection of USA300/Eno‐Antares2. We confirmed the success of the pre‐exposure model by detecting the anti‐Hla IgG (Figure S5F). Results demonstrated that in this pre‐exposure model, Hm3‐4 effectively overcame immune imprinting interference, significantly enhanced host bacterial clearance capacity, and ultimately increased the protection rate to 75% (Figure S5G–I).

### 2.6 Hm3‐4 Synergistically Neutralizes Hla and Attenuates Inflammation in *S. aureus* Pneumonia

*S. aureus* pneumonia, encompassing hospital‐acquired and ventilator‐associated forms, carries a mortality rate of approximately 60% [53, 54, 55]. Key pathological features include alveolar consolidation driven by neutrophil‐dominated inflammation, extracellular matrix degradation leading to alveolar destruction [25, 56, 57, 58], and elevated pulmonary protein levels resulting from increased vascular permeability [25, 56, 59, 60].

Hla is a major virulence factor in *S. aureus* pneumonia. It mediates epithelial and endothelial injury via ADAM10‐dependent E‐cadherin cleavage and synergistically activates the nucleotide‐binding domain, leucine‐rich repeat, and pyrin domain‐containing protein 3 (NLRP3) inflammasome, promoting IL‐1β production and exacerbating disease severity [61, 62, 63, 64, 65, 66, 67].

**FIGURE 6:**
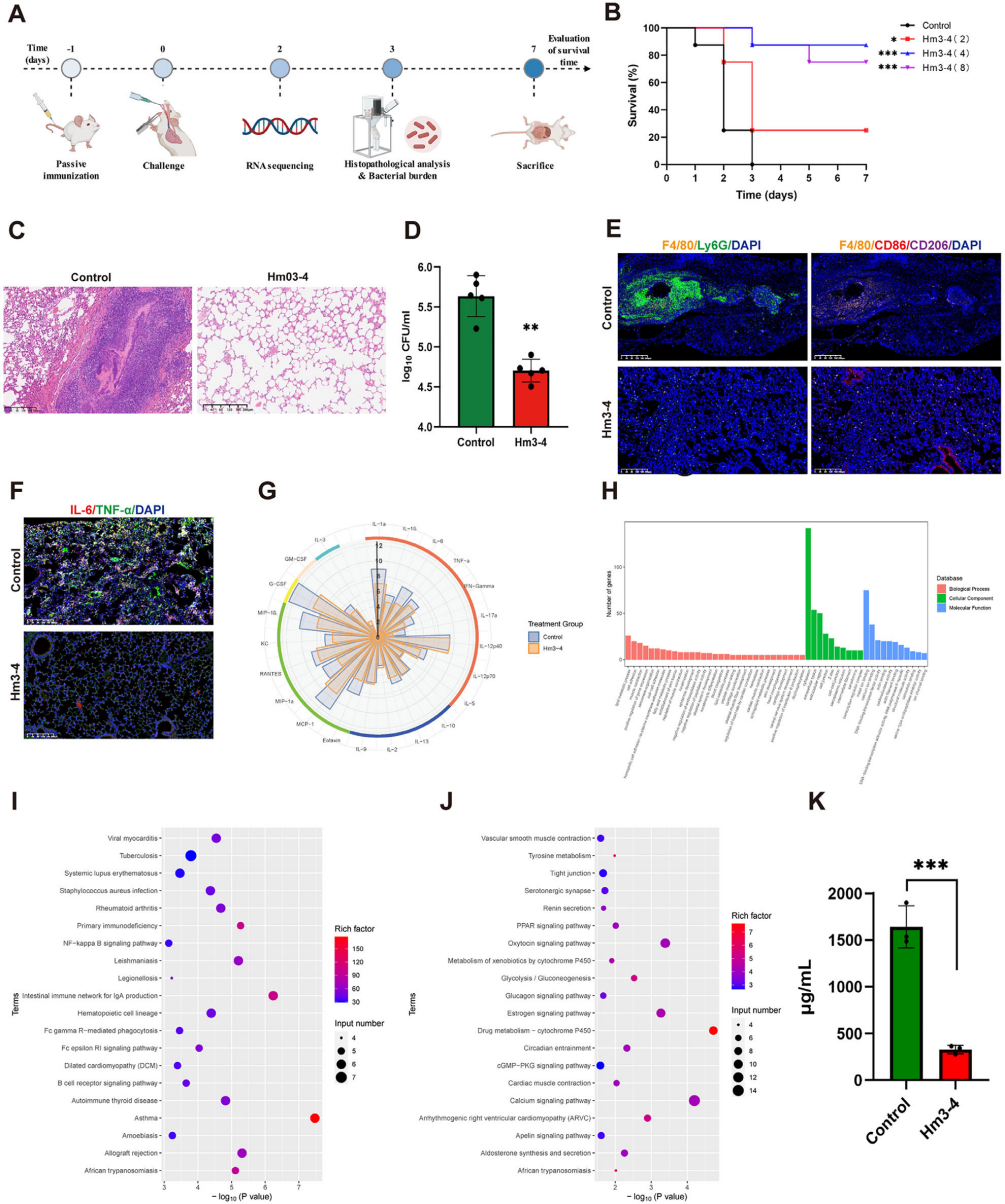
The protective efficacy of a dual‐antibody cocktail against *S. aureus* pneumonia. (A) The immunization, challenge, and detection timelines are shown. Mice were immunized with Hm3‐4 24 h prior to *S. aureus* challenge via intratracheal instillation. The lung tissue was stained with H&E 72 h postinfection, and the bacterial burden was also quantified. Survival rate of the mice was monitored for 7 days. (B) The protective efficacy of the mAbs in mice challenged with a lethal dose of USA300. (C) Histopathologic evaluation of the lung 72 h postinfection. (D) The bacterial burden in the lung was determined. (E, F) Immunofluorescence labeling of cell markers (E), including F4/80, Ly6g, CD86, and CD206, and cytokines (F), including IL‐1β and IL‐6, in the lung. (G) Cytokine analysis in lung tissue was conducted using Luminex. (H–J) RNA‐seq assay of lung tissue was performed 48 h postinfection. GO enrichment of downregulated genes (H); KEGG enrichment of upregulated (I) and downregulated (J) genes. (K) The total protein concentration in BALF was detected using ELISA. *n* = 8 (B), *n* = 3 (D, H–K), and *n* = 4 (G) per group. Data are presented as mean ± SD and analyzed using GraphPad Prism software (v.10.1.2). *p*‐values were determined by simple survival analysis (Kaplan–Meier) in (B); *p*‐values were determined by unpaired *t*‐test in (D, K). *p < 0.05; **p < 0.01; ***p < 0.001.

To evaluate the protective efficacy of the dual‐antibody cocktail, BALB/c mice received Hm3‐4 (20, 40, or 80 mg/kg) or c‐IgG (40 mg/kg) intravenously 24 h before intratracheal challenge with MRSA USA300 (Figure 6A). Hm3‐4 significantly improved 7‐day survival, with the 40 mg/kg dose providing optimal protection (Figure 6B). Lung pathology and bacterial burden were assessed at 72 h postinfection. Histopathological analysis showed that c‐IgG‐treated mice developed severe alveolar inflammation characterized by edema, hemorrhage, and diffuse neutrophil infiltration. In contrast, Hm3‐4‐treated mice exhibited mild to moderate inflammation, localized neutrophil accumulation, and preservation of alveolar architecture (Figure 6C). Lung bacterial loads were significantly reduced in Hm3‐4‐treated mice compared with c‐IgG controls (Figure 6D). Notably, no bacteria were detected in the kidneys of Hm3‐4‐treated mice (data not shown), indicating effective local infection control and prevention of systemic dissemination.

To elucidate the underlying protective mechanisms, we performed multilevel analyses of lung tissue: (1) Immune cell profiling: Neutrophils were the predominant infiltrating cell type. CD86 expression was significantly reduced in lungs from Hm3‐4‐treated mice, consistent with findings in the sepsis model. Because macrophages were present at low abundance, no statistically significant differences in macrophage subtype distribution were detected (Figure 6E). (2) Transcriptomic analysis: RNA sequencing identified 383 downregulated and 30 upregulated genes in lungs from Hm3‐4‐treated mice. Downregulated genes were significantly enriched in calcium ion (Ca^2+^) binding (Gene Ontology [GO]:0005509, *p* = 1.25 × 10^−10^), calmodulin binding (GO:0005516, false discovery rate [FDR] = 0.008), and the Ca^2+^ signaling pathway (Kyoto Encyclopedia of Genes and Genomes [KEGG], *p* = 6.34 × 10^−5^). Upregulated genes were enriched in Fcγ receptor‐mediated phagocytosis (KEGG, *p* = 0.00035) (Figure 6H–J). (3) Signaling pathways: Hm3‐4 treatment suppressed Ca^2+^ influx, thereby limiting NLRP3 inflammasome activation and nuclear factor‐κB (NF‐κB) signaling. This was associated with decreased expression of key pro‐inflammatory cytokines, including IL‐6 and IL‐1β (Figure 6F). Concurrently, enhanced Fcγ receptor‐mediated phagocytosis likely contributed to improved pathogen clearance. (4) Cytokine profiling: Expression of 16 pro‐inflammatory cytokines, including IL‐1β, TNF‐α, and IL‐6, was significantly reduced in lung tissue from Hm3‐4‐treated mice (Figure 6G; analytes below the detection limit not shown). (5) Vascular permeability: Total protein levels in bronchoalveolar lavage fluid (BALF) were significantly lower in Hm3‐4‐treated mice compared with controls (Figure 6K), indicating preservation of vascular integrity.

Collectively, these findings demonstrate that Hm3‐4 mitigates the pathological hallmarks of *S. aureus* pneumonia through synergistic neutralization of Hla and coordinated suppression of inflammatory signaling pathways, while simultaneously enhancing Fcγ receptor‐mediated pathogen clearance.

### 2.7 Antibody–Antibiotic Synergy Enhances Therapeutic Efficacy in *S. aureus* Sepsis

Our findings demonstrate that prophylactic administration of the dual‐antibody cocktail Hm3‐4 reduced disease severity in *S. aureus* sepsis and pneumonia models, improved survival, and conferred protection against diverse clinical isolates. However, treatment of *S. aureus* infections remains clinically challenging, even for antibiotic‐susceptible strains [68, 69, 70]. We therefore evaluated the therapeutic efficacy of intravenous Hm3‐4 in combination with low‐dose intraperitoneal vancomycin or linezolid in a murine sepsis model.

**FIGURE 7:**
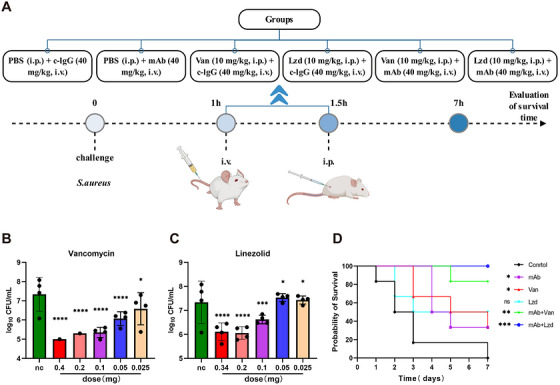
Synergistic effect of the antibody‐antibiotic combination on *S. aureus* sepsis. (A) The challenge, immunization, and monitoring timelines are shown. Mice were challenged with *S. aureus* via i.v. injection. Subsequently, mice were injected with Hm3‐4 (i.v.) combined with vancomycin (Van) or linezolid (Lzd) (i.p.) 1 h after the challenge. (B, C) One hour postinfection, various concentrations of antibiotics were administered intraperitoneally to ascertain the minimal effective therapeutic dose for the two antibiotics. *n* = 5 mice. (D) The therapeutic efficacy of Hm3‐4 combined with low‐dose vancomycin or linezolid against a lethal‐dose challenge with USA300. *n* = 10 mice. Data are presented as mean ± SD and analyzed using GraphPad Prism software (v.10.1.2). *p*‐values were determined by ordinary one‐way ANOVA with Dunnett's multiple comparison in (B) and (C); *p*‐values were determined by simple survival analysis (Kaplan–Meier) in (D). ns, not significant; *p < 0.05; **p < 0.01; ***p < 0.001; ****p < 0.0001.

All treatments were initiated 1 h after intravenous infection (Figure 7A). Combination therapy consisting of Hm3‐4 (40 mg/kg) plus low‐dose vancomycin (5 mg/kg) or linezolid (5 mg/kg) significantly improved survival compared with either antibiotic or Hm3‐4 monotherapy (Figure 7B–D). Hm3‐4 monotherapy alone delayed mortality by approximately 3 days relative to controls; however, by the end of the 7‐day observation period, overall survival did not differ significantly between Hm3‐4‐treated and control groups. This likely reflects rapid bacterial proliferation and systemic dissemination before antibody‐mediated control when therapy is initiated after infection.

Collectively, these results demonstrate that Hm3‐4 synergizes with conventional antibiotics to enhance therapeutic efficacy in established *S. aureus* sepsis. The capacity of Hm3‐4 to potentiate antibiotic activity suggests a strategy to improve clinical outcomes while potentially reducing antibiotic dose requirements and selective pressure for resistance.

## 3 Discussion

*S. aureus*, a major human commensal and opportunistic pathogen, remains a substantial public health threat owing to its adaptability and widespread antimicrobial resistance. Targeting key virulence factors rather than bacterial viability represents a promising strategy to mitigate host damage while reducing selective pressure for resistance. Building on our previous work, we developed Hm3‐4, a dual‐antibody cocktail directed against Hla. Integrated structural, functional, and in vivo analyses demonstrated that although Hm0411 and Hm0399 recognize overlapping epitopes on Hla, they operate through distinct yet complementary mechanisms. Hm0411 forms a large interaction interface and is stabilized by an extensive salt‐bridge network, enabling high‐affinity binding that blocks Hla engagement with its receptor ADAM10 and neutralizes the toxin's cytolytic and prothrombotic activities. In contrast, Hm0399 engages Hla primarily through hydrogen‐bond interactions, resulting in a faster dissociation rate (*K*_off_ = 1.15 × 10^−3^ s**^−1^**). This kinetic profile facilitates monovalent binding of the antigen, which more effectively promotes Fc‐mediated effector functions, thereby boosting immune clearance [50]. The resulting “toxin neutralization (Hm0411)‐immune clearance (Hm0399)” synergy represents a mechanistically integrated strategy distinct from conventional monoclonal antibody cocktails [71]. Importantly, both antibodies retain high‐affinity binding to the fully assembled Hla‐heptamer, indicating that Hm3‐4 can target both soluble monomeric toxin and membrane‐associated heptameric pores, thereby providing comprehensive protection against toxin‐mediated cytopathology.

Hla is a central driver of platelet aggregation, coagulopathy, and multi‐organ injury in *S. aureus* sepsis. Unlike bactericidal therapies, Hla‐neutralizing antibodies do not interfere with early bacterial uptake but instead disrupt downstream toxin‐mediated thrombosis [24, 72, 73]. In the USA300‐induced sepsis model, Hm3‐4 demonstrated a dual mechanism of action. The Hm0411 rapidly neutralized circulating Hla, thereby preventing endothelial and platelet damage. Concurrently, the Hm0399, despite lacking direct Hla‐neutralizing activity in vitro, enhanced the clearance of both Hla‐damaged host cells and free toxin via Fc‐mediated effector functions. We speculate that this dual blockade—combining direct neutralization with Fc‐mediated clearance—synergistically mitigates pathological thrombosis, improves microcirculatory perfusion, and facilitates platelet recruitment to infection sites. Consequently, neutrophil influx and macrophage bactericidal activity are amplified, effectively containing local infection foci and suppressing systemic bacterial dissemination. Ultimately, this dual mechanism restores host immune homeostasis, attenuates disease severity, and significantly improves survival. Notably, Hm3‐4 exhibited broad‐spectrum efficacy. Its protective effect was independent of *S. aureus* genetic background or antibiotic resistance profile, providing robust protection against multidrug‐resistant strains. Critically, in a pre‐exposure model designed to better approximate human colonization history, this dual mechanism effectively counteracted immune imprinting from prior exposure, conferring robust protection against *S. aureus* reinfection.

In *S. aureus* pneumonia, Hla disrupts the alveolar barrier integrity through ADAM10‐dependent E‐cadherin cleavage, NLRP3 inflammasome activation, and Ca^2+^‐dependent cytotoxicity [74]. Hm3‐4 counteracted these processes through coordinated mechanisms. Hm0411 blocked Hla‐ADAM10 interactions, reduced Ca^2+^ influx, and suppressed NLRP3 and NF‐κB signaling, thereby limiting production of pro‐inflammatory cytokines such as IL‐6, IL‐1β, and TNF‐α. Concurrently, Hm0399 enhanced Fcγ receptor‐mediated phagocytic pathways, improving pathogen clearance. Together, these actions reduced inflammatory injury, preserved alveolar architecture, and prevented systemic dissemination, illustrating how dual targeting of toxin activity and immune clearance can disrupt the cycle of tissue damage and immune dysregulation that characterizes severe pneumonia.

Management of invasive *S. aureus* infection remains challenging due to antibiotic resistance, treatment‐related toxicity, and toxin‐mediated immune dysregulation. Prior anti‐Hla antibody strategies have primarily focused on pneumonia [75, 76, 77], whereas Hm3‐4 demonstrated efficacy across both sepsis and pneumonia models. The USA300 strain is a prototypical CA‐MRSA lineage responsible for outbreaks in both community and healthcare settings, particularly in Europe and the United States. In this study, we evaluated the therapeutic efficacy of Hm3‐4 combined with low‐dose vancomycin or linezolid in a sepsis model induced by USA300‐FPR3757. The results demonstrated that the combination therapy significantly enhanced survival rates to over 80%. This synergistic effect likely arises from complementary mechanisms of action: antibiotics effectively reduce bacterial burden, while Hm3‐4 neutralizes toxins and enhances host immune clearance capabilities. This dual strategy not only holds promise for improved therapeutic outcomes but also mitigates risks associated with long‐term, high‐dose antibiotic regimens, such as renal toxicity, and potentially delays the emergence of drug resistance. Given that the Hm0399 antibody component within Hm3‐4 has been shown to effectively activate Fc‐mediated effector functions, future research may explore its development as an Antibody‐drug conjugate (ADC). This approach aims to enhance endocytosis by phagocytic cells (e.g., hepatic Kupffer cells), targeting and eliminating the reservoir of *S. aureus* persisting within them. This is critical because, although studies indicate Kupffer cells can clear approximately 90% of invading bacteria, the remaining ∼10% can secrete sufficient Hla within 4–8 h to lyse host cells and escape, leading to recurrent infection [23]. The core mechanism of ADC therapy is to disrupt this escape pathway at its source through targeted intracellular drug delivery, thereby achieving more comprehensive pathogen eradication.

Taken together, Hm3‐4 represents a dual‐mechanism immunotherapy that targets *S. aureus* pathogenesis at multiple levels. Its advantages include: (i) structurally informed design enabling functional complementarity through distinct binding modes, (ii) integrated inhibition of toxin activity with restoration of immune homeostasis and tissue integrity, and (iii) translational potential through combination with existing antibiotics. These findings support a broader framework for antibody‐based therapies that target both virulence factors and host immune responses.

### 3.1 Limitations

This study has some limitations. First, although the murine pre‐exposure model approximates repeated human exposure to *S. aureus*, the route and timing of infection do not fully replicate natural colonization and disease progression in humans. Second, antibody dosing and pharmacokinetics in mice may not directly translate to humans and will require careful optimization in clinical settings. Third, while the data support synergy between toxin neutralization and Fc‐mediated clearance, additional studies are needed to define the relative contributions of specific Fcγ receptor pathways and to assess long‐term safety and efficacy in larger animal models.

## 4 Conclusions

In summary, we establish a dual‐antibody cocktail strategy that integrates virulence neutralization with enhancement of host immune clearance to counter invasive *S. aureus* infection. By simultaneously targeting toxin activity and immune dysfunction, Hm3‐4 provides broad protection in models of sepsis and pneumonia and synergizes with conventional antibiotics. These findings offer a conceptual framework for next‐generation anti‐virulence immunotherapies aimed at addressing the growing global threat of antimicrobial resistance.

## 5 Materials and Methods

Bacterial strains, plasmids, and key reagents are listed in the Supporting Information Materials.

### 5.1 Animals

Female BALB/c mice (6–8 weeks old, ∼20g) were purchased from Beijing Vital River Laboratory Animal Technology Co., Ltd. (Beijing, China). Mice were housed under specific pathogen‐free conditions with ad libitum access to sterilized food and autoclaved water (121°C). All invasive procedures were performed under sodium pentobarbital anesthesia to minimize discomfort.

### 5.2 Production, Purification, and Characterization of Proteins

Details of recombinant protein production, purification, and characterization are provided in the Supporting Information Materials.

### 5.3 In Vitro Neutralization Assays

Methods for hemolysis inhibition, cytotoxicity inhibition, and assessment of antibody‐mediated blockade of Hla binding to host cells by flow cytometry and LSCM are described in the Supporting Information Materials.

### 5.4 Whole‐Blood Killing Assay

Whole‐blood bactericidal assays were performed in 96‐well round‐bottom microplates. First, opsonization buffer B (OBB) was freshly prepared by mixing 80 mL sterile water, 10 mL 10× Hank's balanced salt solution, 10 mL 1% gelatin, and 5.3 mL fetal bovine serum (FBS). For the assay, each reaction well contained 20 µL antibody from each serum sample, 10 µL *S. aureus* suspension (5 × 10^4^ CFU/mL) diluted in OBB, and 50 µL anticoagulated mouse whole blood. Control wells were set up with an identical total reaction volume, replacing the test antibody with a matched control antibody. After incubation at 37°C for 45 min, 10 µL aliquots of each reaction mixture were collected and spread onto agar plates. Colony‐forming units (CFU) were counted after overnight incubation at 37°C using an automated colony counter (Synbiosis).

### 5.5 Evaluation of In Vivo Protective Efficacy

#### 5.5.1 Toxin Neutralization Model

To determine the minimum lethal dose, recombinant Hla was administered intravenously at doses of 0.125–4 µg per mouse. Thirty minutes later, mice received intravenous Hm0399 or Hm0411 (1–4 µg per mouse) for therapeutic evaluation.

#### 5.5.2 Bacterial Infection Models

Clinical isolates of *S. aureus* were cultured in TSB to mid‐log phase (OD_600_ ≈ 0.8), serially diluted, and administered either intravenously (100 µL) or intratracheally (20 µL).

For prophylactic experiments, monoclonal antibodies (mAbs), including control IgG (c‐IgG) and S283, were administered intravenously 24 h before bacterial challenge. For therapeutic experiments, mAbs and antibiotics were administered 1 h after infection. Unless otherwise specified, 800 µg mAb in 100 µL phosphate‐buffered saline (PBS) was injected intravenously. Vancomycin or linezolid was administered intraperitoneally at 1 mg/kg in 100 µL PBS (prepared at 1 mg/mL). Control animals received PBS or c‐IgG. At designated time points, mice were euthanized, and tissues were collected for analysis of bacterial burden, histopathology, cytokine levels, and transcriptomic profiling. Detailed protocols for bacterial quantification, hematoxylin and eosin (H&E) staining, multiplex immunofluorescence, cytokine analysis, RNA sequencing, and BALF protein measurements are provided in the Supporting Information Materials. Survival was monitored for up to 7 days after infection.

#### 5.5.3 In Vivo Bioluminescence Imaging

Mice were administered Hm3‐4 or c‐IgG intravenously 24 h before intraperitoneal infection with USA300/Eno‐Antares2. Mice were shaved ventrally to reduce signal attenuation. Under anesthesia, HFZ substrate (100 µL) was injected intraperitoneally. Bioluminescence imaging was performed using an in vivo imaging system (IVIS; Xenogen). Imaging was conducted daily for 5 days. Signal intensity was quantified as photons per second per steradian. The red spectrum indicates a stronger signal intensity, while the blue spectrum indicates a weaker signal intensity.

### 5.6 Pre‐Exposure Model

To model prior *S. aureus* exposure, mice were randomized to pre‐exposure or naïve groups and injected intraperitoneally three times at 7‐day intervals with PBS or 1 × 10^7^ colony forming units (CFU) USA300/Eno‐Antares2. Mice then received intravenous Hm3‐4 or PBS on days 5 and 7 after the final exposure. Anti‐Hla IgG titers were measured using ELISA. Seven days after the final exposure, mice were challenged intravenously (2 × 10^9^ CFU) or intraperitoneally (2 × 10^8^ CFU). Survival was monitored, and bacterial burden was assessed by bioluminescence imaging following intraperitoneal injection of L‐hydrofurimazine (100 µL, 3 mM; MedChemExpress, HY‐D1279) under isoflurane anesthesia.

### 5.7 Cryo‐Electron Microscopy (Cryo‐EM) Structure Determination

Purified Hm0399 and Hm0411 in PBS supplemented with 2 mM EDTA and 10 mM freshly prepared L‐cysteine hydrochloride, then adjusted to a final concentration of 1 mg/mL. Papain was added to the solution at an enzyme‐to‐substrate ratio of 1:100 (w/w). The reaction mixture was incubated at 37°C for 3 h, and the digestion was terminated by adding iodoacetamide to a final concentration of 30 mM, followed by a 15 min incubation at room temperature in the dark. The digested products were buffer‐exchanged into PBS (pH 7.4) using 30 kDa molecular weight cut‐off (MWCO) centrifugal filters. The purified Hla protein was incubated at 4°C with Fab0411 or Fab0399 antibody fragments at an optimized molar ratio and subsequently vitrified for cryo‐EM analysis. Statistical parameters for data collection, refinement, and validation are summarized in Table S3, and intermolecular interaction distances are listed in Table S2. Preparation and purification of antibody Fab fragments are described in the Supporting Information Materials.

### 5.8 Measurement of ADCC and ADCP

#### 5.8.1 ADCC and ADCP Reporter Assays

Antibody‐dependent cellular cytotoxicity (ADCC) and antibody‐dependent cellular phagocytosis (ADCP) were assessed using Hla‐expressing HEK293 target cells and control HEK293 cells (RRID: CVCL_0063). Target cells were plated 16–24 h before the assay, trypsinized, centrifuged at 500×*g* for 5 min, and resuspended in fresh medium. Cells (100 µL per well) were seeded into culture plates and incubated overnight at 37°C. Prediluted antibodies were added and incubated with target cells for 1 h at 37°C. Jurkat‐FcγRIIIa effector cells (for ADCC; GM, GM‐C05619, RRID: CVCL_E4JE) or Jurkat‐FcγRIIa effector cells (for ADCP; GM, GM‐C09467) were then added and co‐incubated for 6 h. Luciferase activity was measured using a GM One‐Step Luciferase Reporter Assay Kit (GM‐040503; Genomeditech, Shanghai, China) according to the manufacturer's instructions. Construction of the HEK293‐Hla cell line is described in the Supporting Information Materials.

#### 5.8.2 Phagocytosis of Antigen–Antibody Complexes

RAW264.7 and MHS macrophage cell lines were seeded in glass‐bottom dishes (801001; NEST, Shanghai, China) at 3 × 10^5^ cells per dish and cultured overnight. Cy5‐labeled Hla (2 µg) was preincubated with FITC‐Hm0399, FITC‐Hm0411, or PBS at 37°C for 30 min and added to the cells. After 30 min of incubation, cells were washed three times with PBS. Nuclei were stained with 4′,6‐diamidino‐2‐phenylindole (10 µg/mL; C0060, Solarbio, China) for 5 min. Cells were washed again and imaged using LSCM.

### 5.9 Statistical Analysis

Data are presented as mean ± standard deviation. Statistical analyses were performed using GraphPad Prism version 10.0 (GraphPad Software, San Diego, CA, USA). Continuous variables from two independent cohorts were compared via an unpaired Student's *t*‐test after confirming normality assumptions by the Shapiro–Wilk test and homogeneity of variances by Levene's test. For intergroup comparisons involving three or more experimental conditions, statistical differences were determined using one‐way analysis of variance with Dunnett's post hoc correction. Significance thresholds were established as follows: *p < 0.05, **p < 0.01, ***p < 0.001, ****p < 0.0001.

## Author Contributions

**Liqun Zhao**: methodology, validation, software, investigation, and writing – original draft. **Zhen Song**: validation, investigation, and writing – original draft. **Hongyin Fan**: software and data curation. **Lei Wang**: software and writing – review and editing. **Yun Yang**: validation. **Haiming Jing**: validation. **Xin Xia**: validation. **Yu Wang**: validation. **Leilei Feng**: software. **Sheng Wang**: validation. **Zhifu Chen**: validation. **Qiang Gou**: validation. **Yue Yuan**: validation. **Jinyong Zhang**: validation. **Quanming Zou**: validation. **Hao Zeng**: Supervision, project administration, and funding acquisition. All authors have read and approved the final manuscript.

## Funding

This work was supported by the National Key Research and Development Program of China (2024YFC2310802), the National Natural Science Foundation of China (32270989), and the Natural Science Foundation of Chongqing (cstc2021jcyj‐msxmX1188).

## Ethics Statement

All animal experiments were conducted in accordance with the International Guiding Principles for the Care and Use of Laboratory Animals and were approved by the Animal Welfare and Ethics Committee of the Third Military Medical University (approval no. AMUWEC20223334). Human sample collection and related studies were approved by the Ethics Committee of Jiangsu Provincial Center for Disease Prevention and Control: JSJK2015‐A023‐02. The associated clinical trials were registered at ClinicalTrials.gov under identifiers NCT02804711 and NCT03966040.

## Conflicts of Interest

The authors declare no conflicts of interest.

## Supporting information



Figure S1. The original uncropped full blot.
**Figure S2. Flowchart illustrating the structure deposition of the Hla/Fab 411 complex**.
**Figure S3**. Cryo‐EM and local map of the binding interface of Hla/Fab 411 complex(A), Cryo‐EM and local map of the Hla/Fab399 complex(B). Residues are shown as sticks with oxygen colored in red, nitrogen colored in blue, and sulfur colored in yellow. Colors for Hla are shown in purple.
**Figure S4. The ADCC, ADCP detection, and efficacy inhibiting assay. (A, B**) A549 cells were treated with Hla and added with Hm0399 or Hm0411 together with effector cells, and then ADCC (A) and ADCP (B) were detected using GMOne‐Step Luc assay. (**C, D**) The construction of a lentivirus expression plasmid encoding Hla and confirming detection. The gene information of Hla expression plasmid PGMLV‐CMV‐wHla(Codon opt)‐G4S3 linker‐SS1‐EF1‐ZsGreen1‐T2A‐Puro (C). The infection of the lentivirus vector into HEK‐293 cells was observed by LSCM (D). (**E, F**) The overexpression effect of the gene in HEK‐293 cells infected with lentivirus was determined by flow cytometry. The gate strategy (E). The expression levels of Hla on HEK‐293 cells were detected by Hm0399 and Hm0411 (F). (**G–I**) The efficacy inhibiting assay. The neutralizing activity of Hm0411 with Hm0399 existing (G). The ADCC (H) and ADCP (I) of Hm0399 with Hm0411 existing. Data are presented as mean ± SD and analyzed using GraphPad Prism software (v.10.1.2). Data processing and fitting were performed using variable slope (four parameters) in (A), (B), (H), and (I). *p*‐values were determined by ordinary one‐way ANOVA with Dunnett's multiple comparison in (G). ns, not significant.
**Figure S5. The in vivo protective efficacy of Hm3‐4. (A**) The protective efficacy when extending the observation period to 15 days. (B–E) The protective efficacy on different *S. aureus* strains, including Newman (**B**), NCTC 8325‐4 (**C**), ST59 (**D**), and DU1090 (**E**). *n* = 10 mice per group. Data are presented as mean ± SD and analyzed using GraphPad Prism software (v.10.1.2). *p*‐values were determined using simple survival analysis (Kaplan–Meier) in (A–E, G). *p*‐values were determined using ordinary one‐way ANOVA with Dunnett's multiple comparison in (F). *p*‐values were determined using unpaired *t*‐test in I. ns, not significant; *p < 0.05; **p < 0.01; ***p < 0.001; ****p < 0.0001.
**Table S1 Gene sequences of Hm0399 and Hm0411**.
**Table S2 Intermolecular donor‐acceptor distances for hydrogen bonds**.
**Table S3 Statistics for Cryo‐EM data collection, refinement, and validation**.

## Data Availability

The datasets generated and/or analyzed during the current study are available from the corresponding author on reasonable request.
