# Supplementary material for: A Synergistic Dual‐Antibody Cocktail Targeting α‐Toxin Protects Against Invasive Staphylococcus aureus Infection by Neutralizing Virulence and Enhancing Host Defense
Source: MedComm (2020). 2026 Jul 5;7(7):e70830. doi: 10.1002/mco2.70830 (PMC13334222; doi:10.1002/mco2.70830)
Supplement: Supplementary file 1 — Figure S1. The original uncropped full blot. Figure S2. Flowchart illustrating the structure deposition of the Hla/Fab 411 complex. Figure S3. Cryo‐EM and local map of the binding interface of Hla/Fab 411 complex(A), Cryo‐EM and local map of the Hla/Fab399 complex(B). Residues are shown as sticks with oxygen colored in red, nitrogen colored in blue, and sulfur colored in yellow. Colors for Hla are shown in purple. Figure S4. The ADCC, ADCP detection, and efficacy inhibiting assay. (A, B) A549 cells were treated with Hla and added with Hm0399 or Hm0411 together with effector cells, and then ADCC (A) and ADCP (B) were detected using GMOne‐Step Luc assay. (C, D) The construction of a lentivirus expression plasmid encoding Hla and confirming detection. The gene information of Hla expression plasmid PGMLV‐CMV‐wHla(Codon opt)‐G4S3 linker‐SS1‐EF1‐ZsGreen1‐T2A‐Puro (C). The infection of the lentivirus vector into HEK‐293 cells was observed by LSCM (D). (E, F) The overexpression effect of the gene in HEK‐293 cells infected with lentivirus was determined by flow cytometry. The gate strategy (E). The expression levels of Hla on HEK‐293 cells were detected by Hm0399 and Hm0411 (F). (G–I) The efficacy inhibiting assay. The neutralizing activity of Hm0411 with Hm0399 existing (G). The ADCC (H) and ADCP (I) of Hm0399 with Hm0411 existing. Data are presented as mean ± SD and analyzed using GraphPad Prism software (v.10.1.2). Data processing and fitting were performed using variable slope (four parameters) in (A), (B), (H), and (I). p‐values were determined by ordinary one‐way ANOVA with Dunnett's multiple comparison in (G). ns, not significant. Figure S5. The in vivo protective efficacy of Hm3‐4. (A) The protective efficacy when extending the observation period to 15 days. (B–E) The protective efficacy on different S. aureus strains, including Newman (B), NCTC 8325‐4 (C), ST59 (D), and DU1090 (E). n = 10 mice per group. Data are presented as mean ± SD and analyzed using GraphPad Pr [file MCO2-7-e70830-s001.doc]

**A synergistic dual-antibody cocktail targeting α-toxin protects against invasive *Staphylococcus aureus* infection by neutralizing virulence and enhancing host defense**

Liqun Zhao^1,#^, Zhen Song^1,2,#^, Hongyin Fan^1^, Lei Wang^3^, Yun Yang^1^, Haiming Jing^1^, Xin Xia^4^, Yu Wang^1^, Leilei Feng^3^, Sheng Wang^1^, Zhifu Chen^1^, Qiang Gou^1^, Yue Yuan^1^, Jinyong Zhang^1^, Quanming Zou^1^, Hao Zeng^1,5^,*

^1^National Engineering Research Center of Immunological Products, Department of Microbiology and Biochemical Pharmacy, College of Pharmacy and Laboratory Medicine, Third Military Medical University, Chongqing, 400038, China.

^2^Clinical Laboratory Department, Army 954th Hospital, General Hospital of Tibet Military Region, Tibet 856099, China.

^3^CAS Key Laboratory of Infection and Immunity, National Laboratory of Macromolecules, Institute of Biophysics, Chinese Academy of Sciences, Beijing 100101, China.

^4^Research and Development Department, Chengdu Olymvax Biotechnology Co., Ltd., Chengdu, Sichuan 610095, China.

^5^State Key Laboratory of Trauma and Chemical Poisoning, Third Military Medical University, Chongqing 400038, China

^#^ These authors contributed equally.

* Correspondence: Hao Zeng; zeng1109@163.com.

**Materials and Methods**

**Cells and Reagents**

HEK293F cells were obtained from Thermo fisher scientific (Cat＃PTA-5080, RRID: CVCL_6642) and cultured in HEK293 expression medium (OPM Bioscience, 81075-001). The cells were maintained in a humidified incubator at 37°C with 5% CO_2_ and agitated at 120 rpm. MH-S cells (ATCC, RRID: CVCL_3855) and RAW264.7 cells (ATCC, RRID: CVCL_C6XG) were cultured in RPMI-1640 medium supplemented with 10% (v/v) FBS (Hyclone, SH30406.05) and 100 U/mL penicillin/streptomycin solution (Hyclone, SV30010). A549 cells (ATCC, RRID: CVCL_C8WF)) was cultured in F12K medium supplemented with 10% (v/v) FBS (Hyclone, SH30406.05) and 100 U/mL penicillin/streptomycin solution (Hyclone, SV30010)

**Bacteria strains**

MRSA 252, USA300-FPR3757, and Newman strains were procured from the American Type Culture Collection (ATCC, Manassas, VA, USA). NCTC 8325-4 and DU1090 were purchased from the China Medical Culture Collection Center (CMCC). Bacterial cultures were propagated in trypticase soy broth (TSB, AOBOX Biotechnology, Beijing, China) under aerobic conditions (37°C, 220 rpm), with colony-forming unit (CFU) quantification performed on trypticase soy agar (TSA, AOBOX Biotechnology, Beijing, China). For long-term preservation, bacterial stocks were cryopreserved at −80°C in TSB supplemented with 25% (v/v) sterile glycerol. Prior to experimental use, bacterial suspensions were standardized to an optical density (OD600) of 1.0, corresponding to 1×10^9^ CFU/mL as per ATCC quantification guidelines.

**Protein production and purification**

The recombinant proteins Hla, Hm0399 and Hm0411 were produced utilizing either *E. coli* or the HEK293F expression system in our laboratory. Proteins expressed in *E. coli* were purified using Glutathione Sepharose (Cytiva, 17075604), followed by HiLoad™ 16/600 Superdex™ 200 pg (Cytiva, 45002490) for further purification. Hm0399 and Hm0411 expressed in HEK293F cells were purified using Protein A Agarose (Byotime, P2015). The obtained proteins were collected and analyzed by 10% SDS-PAGE and Western blotting.

**Enzyme-linked immunosorbent assay (ELISA)**

First, 96-well ELISA plates (31111B, Labselect, Shanghai, China) were coated with purified antigen in a carbonate-bicarbonate buffer (pH 9.6) using 2 μg/well. After blocking with 5% bovine serum albumin (BSA), the wells were incubated with serum or mAb serially diluted in PBS containing 1% BSA at 37°C for 1 h. Bound antibodies were detected using HRP-conjugated goat anti-human IgG (W4031, Promega).

**Biolayer interferometry (BLI) assay**

The binding affinity between mAbs and purified Hla was analyzed using the Octet RED96 system (ForteBio, Fremont, USA). The mAbs were biotinylated using a Biotinylation Kit (Genemore, G-MM-IGT) at a 3:1 biotin-to-protein molar ratio; successful biotinylation was confirmed using ELISA. Subsequently, the biotinylated antibodies were immobilized onto streptavidin biosensors at appropriate concentrations. Global fitting of the binding kinetics was performed by evaluating the association and dissociation phases of Hla over a suitable range of molar concentrations (31.3 nM to 500 nM). The Octet Analysis Studio 12 software was used to analyze the kinetic responses using a 1:1 model, yielding the Ka, Kdis, and Kd.

**Neutralization assay**

The series of diluted solutions containing Hm0399 or Hm0411 were mixed with 25 ng/well Hla in a 96-well plate (NEST, China) and then incubated at 37°C for 30 min. Subsequently, an equal volume of 1% RBCs was added to each well and incubated at 37°C for an additional 30 min. Each plate included an Hla-only control for cell lysis, and 1% RBCs in an equivalent volume of PBS served as blank. The polyclonal antibody present in the vaccine-immunized serum served as a positive control for neutralization. Subsequently, the plate was centrifuged at 500 × g for 5 min. Then, 100 mm was collected from the top layer of the supernatant, and A540 values were measured using a microplate reader (Bio-Rad, USA). After subtracting background absorbance, the relative hemolysis of each diluted mAb to the Hla-only control was calculated.

To confirm neutralization activity, A549 lung epithelial cells (ATCC, Manassas, USA) were cultivated in DMEM containing 10% FBS and 1% penicillin-streptomycin at 37°C and 5% CO_2_. Then, 2 × 10^4^ A549 cells were seeded into a 96-well plate and incubated for 12–16 h. The Hla sample was incubated with a series of mAbs at varying concentrations for 30 min at 37°C, and was then added to the A549 cells. Cells that were not subjected to the Hla control were designated as live control, and cells that were subjected to the Hla control were designated as an Hla-only control for cell lysis. The polyclonal antibody present in the vaccine-immunized serum served as positive control. Following a 6-h incubation at 37°C, cell viability was detected using a Cell Titer-Glo® 3D Cell Viability Assay Kit (G9681, Promega, USA).

A series of diluted solutions containing Hm0399 at concentrations corresponding to 20‑, 10‑, 1‑, and 0.1‑fold that of Hm0411 were separately mixed with Hm0411 at its EC50 concentration. Subsequently, 25 ng/well of Hla was added, and the mixtures were incubated at 37°C for 30 min. An equal volume of 1% red blood cells (RBCs) was then added to each well, followed by further incubation at 37°C for 30 min. Each plate contained an Hm0399 control for cell lysis, and 1% RBCs in an equivalent volume of PBS served as the blank control. The plate was then centrifuged at 500 × g for 5 min. Next, 100 mm of the upper supernatant was collected, and the absorbance at 540 nm (A540) was measured using a microplate reader (Bio-Rad, USA). Relative hemolysis was calculated after subtraction of the background absorbance.

**Flow cytometry**

The binding of Cy5-Hla to A549 cells was quantitatively analyzed using flow cytometry. First, 2 μg Cy5-Hla was incubated with different concentrations of Hm0411 at 37°C for 30 min. Then, the mixture was added to A549 cells, and the cells were incubated at 37°C for 30 min. Finally, the A549 cells were collected, washed with PBS three times, and analyzed using flow cytometry (BD, Fortessa) to detect Cy5-positive cells. The data was analyzed using FlowJo software (v10.8.1, BD, Kentana, USA).

**Immunolabeling**The Hla protein was fluorescently labeled using CY5-SE (HY-D0819, MCE, New Jersey, USA), following the manufacturer's protocol. Briefly, purified Hla protein was incubated with Cy5 NHS ester (molar ratio 1:20) in 0.1 M sodium bicarbonate buffer (pH 8.3) for 2 h at 25°C in the dark. Unconjugated dye was removed by size-exclusion chromatography using a Sephadex G-25 desalting column equilibrated with PBS (pH 7.4).

Monoclonal antibodies (Hm0399 and Hm0411) were labeled with fluorescein isothiocyanate (FITC) using a FITC antibody labeling kit (BA00111, Bioss, Beijing, China). The antibody solution was adjusted with conjugation buffer (10 μL buffer per 100 μL antibody). Reconstituted FITC was added to the antibody at a ratio of 4.0 μL of FITC per 100 μg of antibody, followed by vortex mixing. The antibody-FITC mixture was incubated on a horizontal shaker (25°C, 60 rpm) for 1 h in the dark. Pre-equilibrated purification columns were centrifuged (3,000 × g, 2 min), and the flow-through buffer was temporarily retained. The antibody-FITC conjugate was loaded onto the column resin. The column was centrifuged (3,000 × g, 2 min) to collect purified conjugates.

**Laser Scanning Confocal Microscopy (LSCM)**

Blocking effects of the mAbs on the binding of Cy5-Hla to A549 cells were observed using LSCM. In brief, 3×10^5^ A549 cells were seeded into a cell-culture dish (801001, NEST, China) and cultured for 24 h. The cells were pre-incubated with the mAbs at 37°C for 30 min and were then treated with 5.5 μg Cy5-Hla. Following a 30-min incubation, the top liquid layer was discarded. The cells were washed three times with PBS and then treated with 1 μM DIO (HY-D0969, MCE, America) for 5 min to stain the cytoskeleton. The cells were then washed three times using PBS. Next, the cells were fixed using 4% paraformaldehyde (PFA) for 15 min, followed by three washes with PBS. Nuclear staining was performed using 10 μg/mL DAPI (C0060, Solarbio, China) for 5 min. After washing three times with PBS, the cells were then observed using LSCM.

**Histopathological analysis**

At the predetermined time points, the kidneys, livers, and lungs were harvested from the mice. The tissues were fixed and embedded in paraffin for the preparation of consecutive sections, which were subsequently stained with hematoxylin and eosin (H&E). The slides were observed using a light microscopy (Eclipse E200, Nikon, Japan), and the inflammatory responses were assessed by professionals.

**Preparation and purification of antibody Fab fragments**

For the Cryo-EM study, purified Hm0399 and Hm0411 underwent digestion with immobilized papain, adhering to the manufacturer's protocol. First, Hm0399 and Hm0411 were dissolved in 0.1 M PBS and subsequently digested in a buffer comprising 0.1 M EDTA and 0.5 M cysteine–HCl (pH 7.0) at 37°C for 30 minutes. The resulting Hm0399 and Hm0411 Fab fragments were purified using protein A affinity chromatography followed by Superdex 200 column gel filtration (Cytiva, 28990944). The purified proteins were then identified via SDS-PAGE and stored at −80°C for subsequent assays.

Hla and Fab0399 or Fab0411 were combined at a molar ratio of 4:1 and incubated for 30 minutes to facilitate complete binding. Subsequently, Hla, Fab0399, Fab0411, and the complexes were applied to a Superdex 200 gel filtration column in phosphate-buffered saline (PBS) (pH 7.2). The elution peaks corresponding to each species were collected and analyzed by 10% SDS-PAGE.

**The ADCC and ADCP assay**

Target cells (A549) were pretreated with 2 μg/mL freshly prepared Hla for 30 minutes. Following pretreatment and washing, Hm0399 or Hm0411 antibodies were added and incubated for 1 hour, after which effector cells were introduced for ADCC/ADCP functional assays. The detection of ADCC and ADCP was used an anti-CLDN18.2 hIgG1 antibody (Zolbetuximab, RRID: AB_3634522) as positive control. 16-24 hours before the experiment, target cells (including Hla-HEK-293 cells and control cells) were retrieved, trypsinized, and subsequently collected by centrifugation. The cell concentration was adjusted using fresh culture medium, and 100 μL/well was dispensed into the culture plate. The plate was then incubated overnight at 37℃ in a humidified incubator with 5% CO2. Afterward, the supernatant was removed, and pre-diluted antibodies were added to the designated wells and incubated for 1 hour under the same conditions. Subsequently, ADCC Jurkat-FcγRIIIa effector cells (GM, GM-C05619, RRID: CVCL_E4JE) or ADCP Jurkat-FcγRIIa effector cells (GM, GM-C09467) were added into the mixtures and co-incubated for 6 hours at 37°C with 5% CO2. All procedures strictly followed the manufacturer's instructions, and the luciferase reporter gene assay was performed according to the protocol provided in the GMOne-Step Luciferase Reporter Gene Assay Kit (GM, GM-040503).

**The construction of lentivirus vector**

The Hla expression plasmid was used by a PGMLV-CMV-wHla (Codon opt)-G4S3 linker-SS1-EF1-ZsGreen1-T2A-Puro and the sequence was determined using the primers of 2H7-seqR (CCAGTACACGACATCACTTTCCC) and H1-3F (CCATTGACGTCAATGGGAG). The sequence was listed:

GCCACCATGAAGACCAGAATCGTGTCTAGCGTGACAACCACCCTGCTGCTCGGCTCTATCCTGATGAACCCTGTGGCCGGCGCCGCCGACTCCGATATCAACATCAAGACTGGAACCACAGATATCGGCAGCAACACAACCGTGAAGACAGGCGATCTGGTCACCTACGACAAAGAGAACGGCATGCACAAGAAAGTGTTCTACAGCTTCATCGACGACAAGAACCACAACAAGAAGATCCTGGTGATTAGAACCAAGGGCACCATCGCCGGCCAGTACCGGGTGTATAGCGAGGAAGGCGCCAACAAAAGCGGACTGGCCTGGCCTAGCGCCTTTAAGGTGCAGCTGCAGCTGCCTGACAACGAGGTGGCCCAGATCAGCGACTACTACCCCAGAAACAGCATTGACACCAAGGAATACATGAGCACACTGACCTATGGATTTAACGGCAACGTGACCGGCGACGATAGCGGCAAGATCGGCGGCCTGATCGGAGCTAATGTGTCCATCGGCCACACCCTGAAGTACGTGCAACCTGATTTCAAGACAATCCTGGAAAGCCCCACCGATAAGAAGGTCGGCTGGAAGGTTATCTTCAACAACATGGTGAACCAGAACTGGGGCCCTTACGACAGAGATAGCTGGAACCCCGTGTACGGAAATCAACTGTTCATGAAAACAAGAAATGGCTCCATGAAAGCCGCTGAAAACTTCCTGGACCCAAACAAGGCTTCTAGCCTGCTGAGCTCCGGCTTCAGCCCTGACTTCGCCACAGTGATCACCATGGACAGAAAGGCCACCAAACAGCAGACCAACATCGACGTGATCTACGAGCGGGTGCGGGACGACTACCAGCTGCATTGGACATCTACAAATTGGAAGGGTACAAACACCAAGGACAAGTGGACCGACCGCAGCTCTGAGAGATACAAGATCGATTGGGAGAAGGAAGAGATGACCAATGGTGGAGGCGGGTCTGGGGGCGGAGGTTCAGGCGGGGGTGGTTCCGAACAGCTTGAAAATGGTGGGACATCCTTATCAGAGAAAACAGTTCTTCTGCTGGTGACTCCATTTCTGGCAGCAGCCTGGAGCCTTCATCCCTAA

Then, high-purity, endotoxin-free lentiviral vectors and helper packaging plasmids were co-transfected into HEK-293T cells (RRID: CVCL_0063) using HG Transgene™ Reagent. Following transfection (10–12 hours), Enhancing Buffer was added to the culture medium. Fresh medium was replaced 8 hours post-buffer addition. The cells were further cultured for 48 hours, after which the lentivirus particle-enriched supernatant was collected and concentrated to obtain high-titer lentiviral stock. Viral titer was quantified and standardized through functional assays in HEK-293T cells.

HEK-293 cells were seeded in a 24-well plate at a density of 5×10⁴ cells per well on the first day. On the second day, prior to infection, the viral stock was thawed on ice after removal from the -80°C freezer. The lentiviral stock was diluted to a multiplicity of infection (MOI) of 10 using 500 μL of complete medium. The existing medium in the treatment groups was aspirated, and 300 μL of lentivirus-containing diluted medium was added to the cells. Sixteen hours post-infection (third day), the lentivirus-containing medium was completely replaced with 500 μL of fresh complete medium.

Infection efficiency was evaluated on the fifth day by observing fluorescence signals under an inverted fluorescence microscope to estimate lentiviral transduction efficiency. For stable cell line selection, puromycin-resistant cells were screened using a lethal concentration of 1.5 μg/mL and a maintenance concentration of 0.75 μg/mL. Two rounds of antibiotic selection (2 days per round, optimized through preliminary antibiotic testing) were performed to establish stable cell lines. Post-selection, cells were maintained in DMEM supplemented with 10% fetal bovine serum (FBS), 1% penicillin-streptomycin (P/S), and 0.75 μg/mL puromycin.

**The determing of overexpression effect of gene in HEK-293 cells infected with lentivirus**

Cell samples were resuspended in 500 μL ice-cold 1% BSA/PBS and centrifuged at 1380 ×g for 1 minute to remove the supernatant. Primary antibodies were diluted as follows: Antibody 3 (11 mg/mL stock) was mixed at 1 μL with 1099 μL 1% BSA/PBS to achieve a 10 μg/mL working concentration, while Antibody 4 (4.5 mg/mL stock) was diluted by combining 1 μL with 449 μL 1% BSA/PBS for a final 10 μg/mL concentration. Cells were incubated with 100 μL diluted primary antibodies at 4°C for 30 minutes under light-protected conditions. Post-incubation, cells were washed twice with 500 μL 1% BSA/PBS, followed by centrifugation at 1380 ×g for 1 minute each. Secondary antibody dilution utilized Allophycocyanin (APC)-conjugated F(ab’)₂ fragment donkey anti-human IgG (H+L) (1 mg/mL stock, RRID: AB_10893271), prepared by mixing 2 μL antibody with 998 μL 1% BSA/PBS to yield a 2 μg/mL working solution. Cells were then incubated with 100 μL diluted secondary antibody under identical conditions (4°C, 30 minutes, light protection) and washed twice as described. Finally, cells were resuspended in 300 μL 1% BSA/PBS, and 100 μL of the suspension was subjected to flow cytometry analysis.

**STR report of the cell line**

The STR reports of A549 cell line, ADCC FcγRIIIa(158V) jurkat effector cell line, and ADCP FcγRIIa jurkat effector cell line were uploaded as supplementary materials.


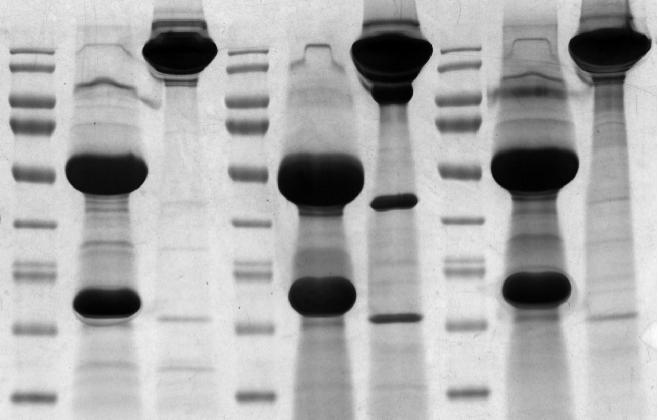


Figure S1. The original uncropped full blot.


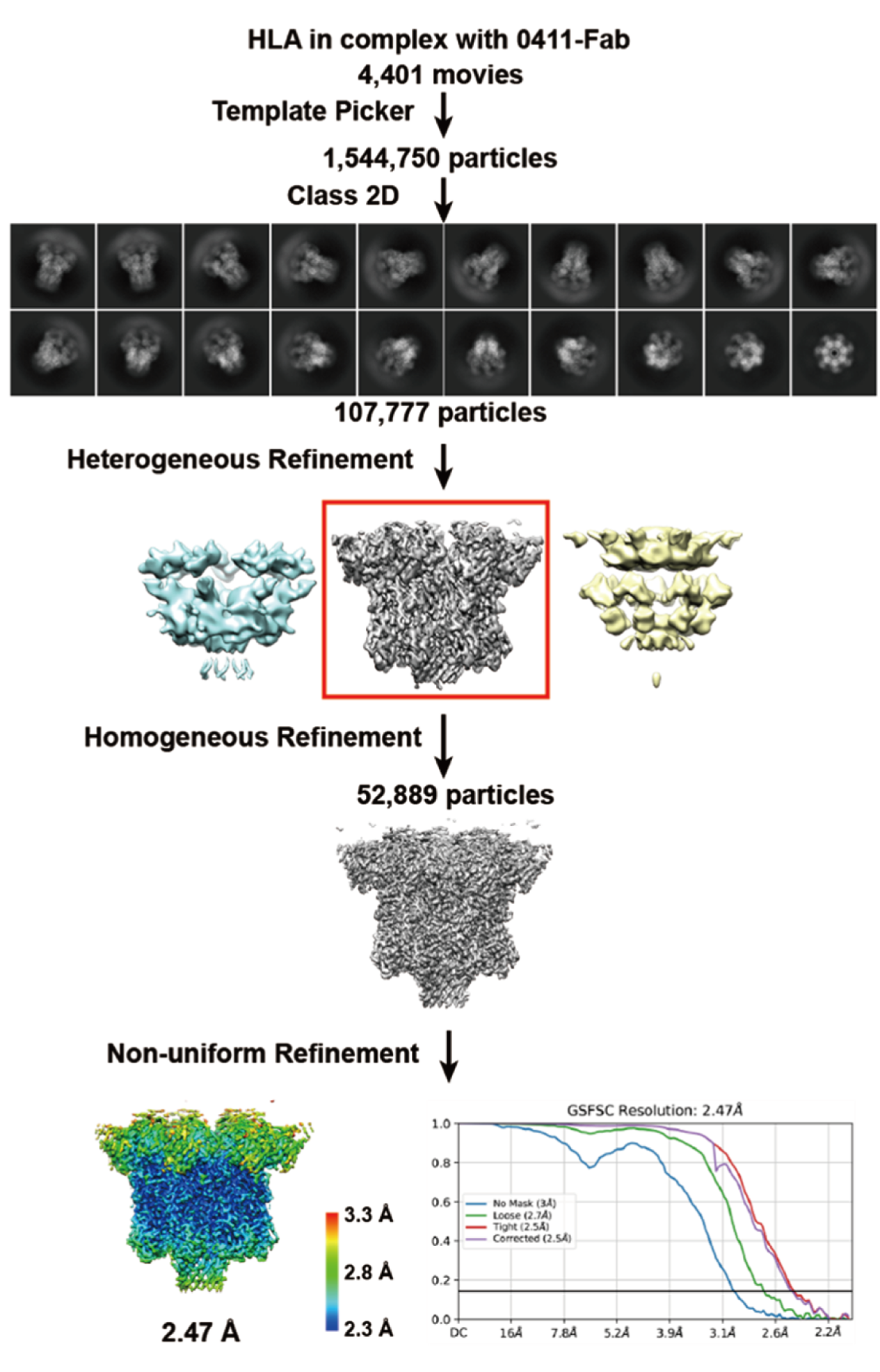


**Figure** **S2. Flowchart illustrating the structure deposition of the Hla/Fab 411 complex**


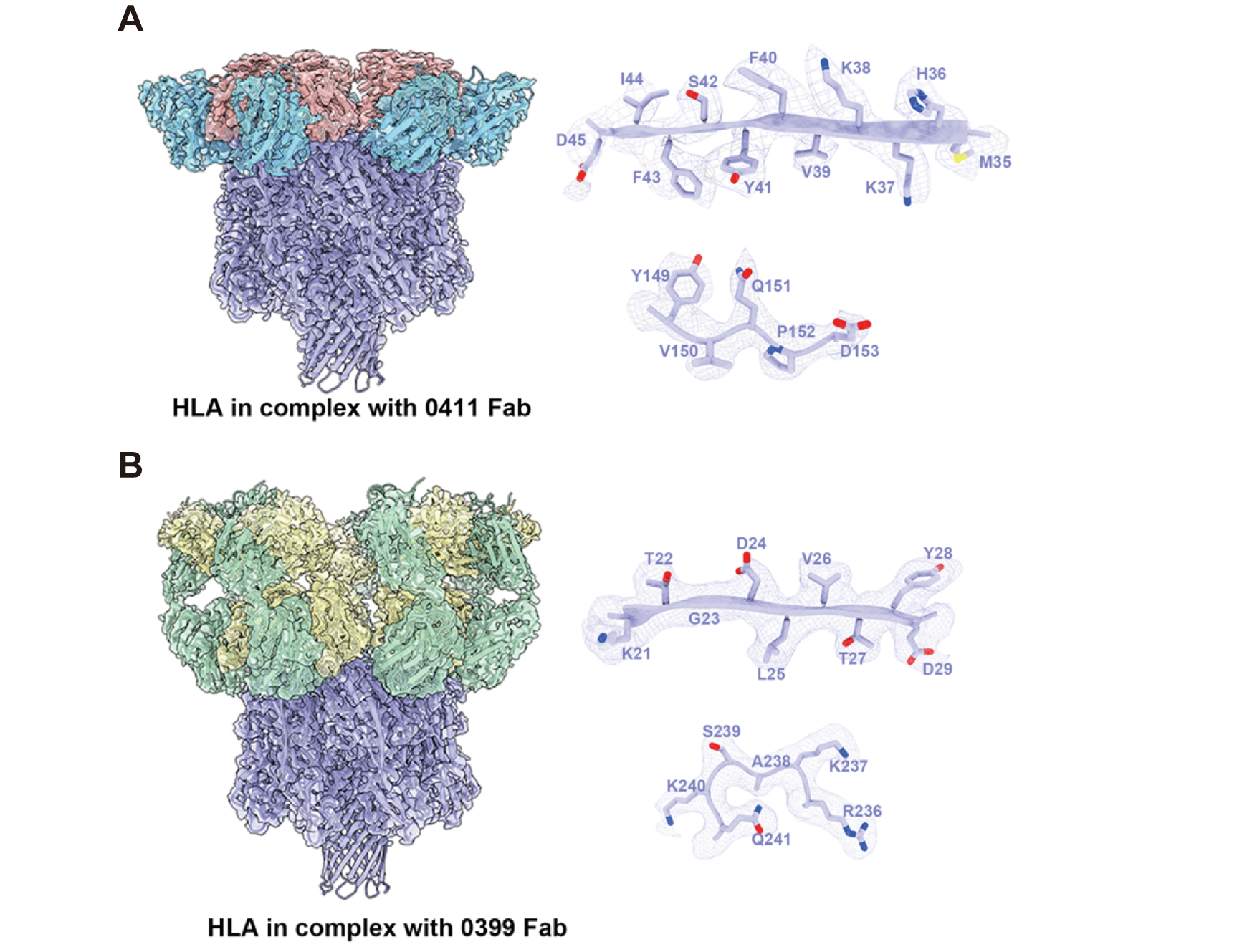


**Figure S3.** Cryo-EM and local map of the binding interface of Hla/Fab 411 complex(A), Cryo-EM and local map of the Hla/Fab399 complex(B). Residues are shown as sticks with oxygen colored in red, nitrogen colored in blue and sulfurs colored in yellow. colors for Hla are shown purple.


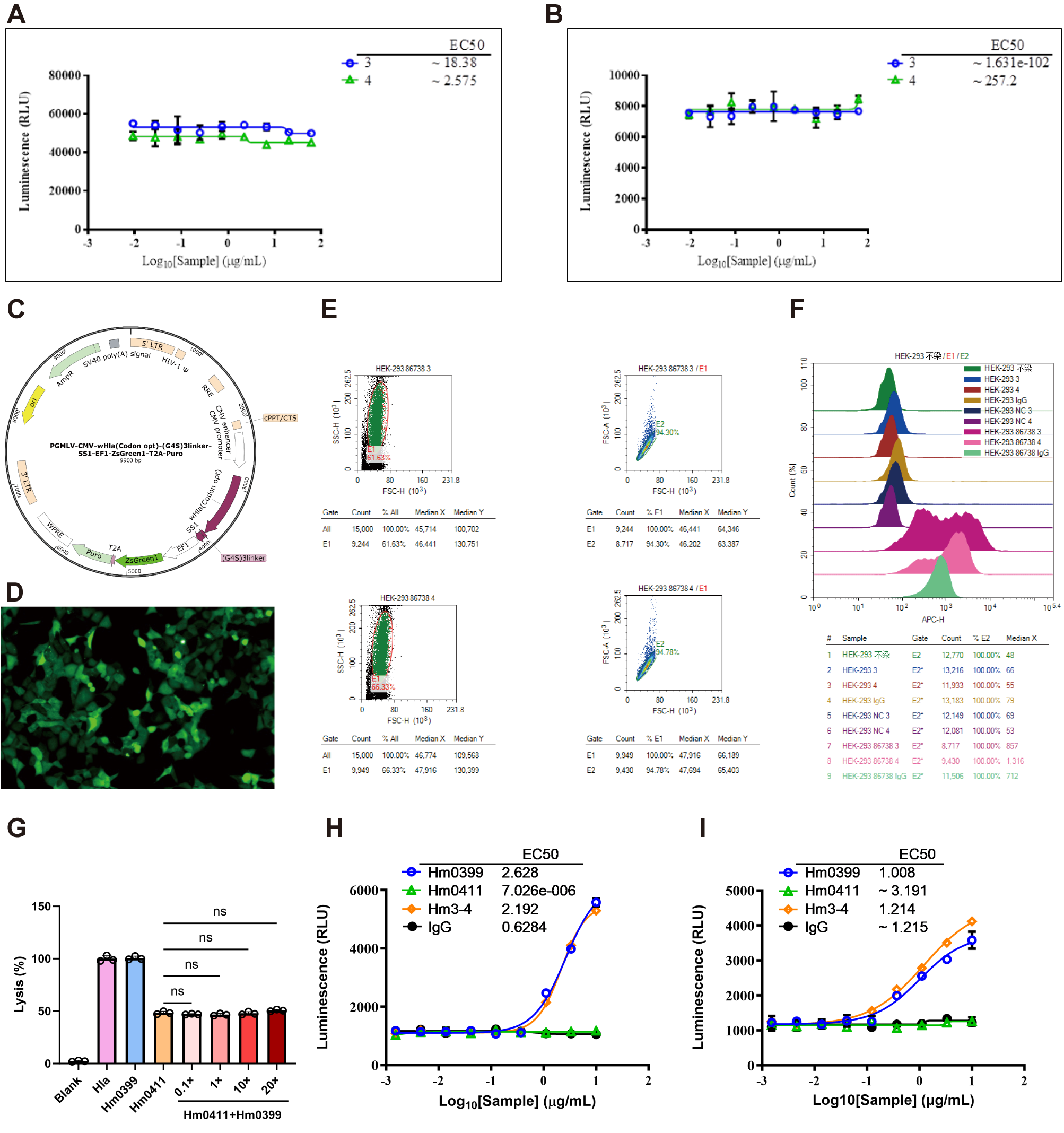


**Figure S4. The ADCC, ADCP dection and efficacy inhibiting assay.** A and B, A549 cells were treated with Hla and added with Hm0399 or Hm0411 together with effector cells, and then ADCC (A) and ADCP (B) were detected using GMOne-Step Luc assay. **C and D,** The construction of lentivirus expression plasmid encoding Hla and confirming detection. The gene information of Hla expression plasmid PGMLV-CMV-wHla(Codon opt)-G4S3 linker-SS1-EF1-ZsGreen1-T2A-Puro (C). The infectious of lentivirus vector to HEK-293 cells was observed by LSCM (D). E and F, The overexpression effect of gene in HEK-293 cells infected with lentivirus was determined by flow cytometry. The gate strategy (E). The expression levels of Hla on HEK-293 cells was detected by Hm0399 and Hm0411 (F). G to I, The efficacy inhibiting assay. The neutralizing activity of Hm0411 with Hm0399 existing (G). The ADCC (H) and ADCP (I) of Hm0399 with Hm0411 existing. Data are presented as mean ± SD and analyzed using GraphPad Prism software (v.10.1.2). Data processing and fitting were performed using variable slope (four parameters) in A, B, H, and I. *P* values were determined by ordinary one-way ANOVA with Dunnett's multiple comparison in G. ns, not significant.


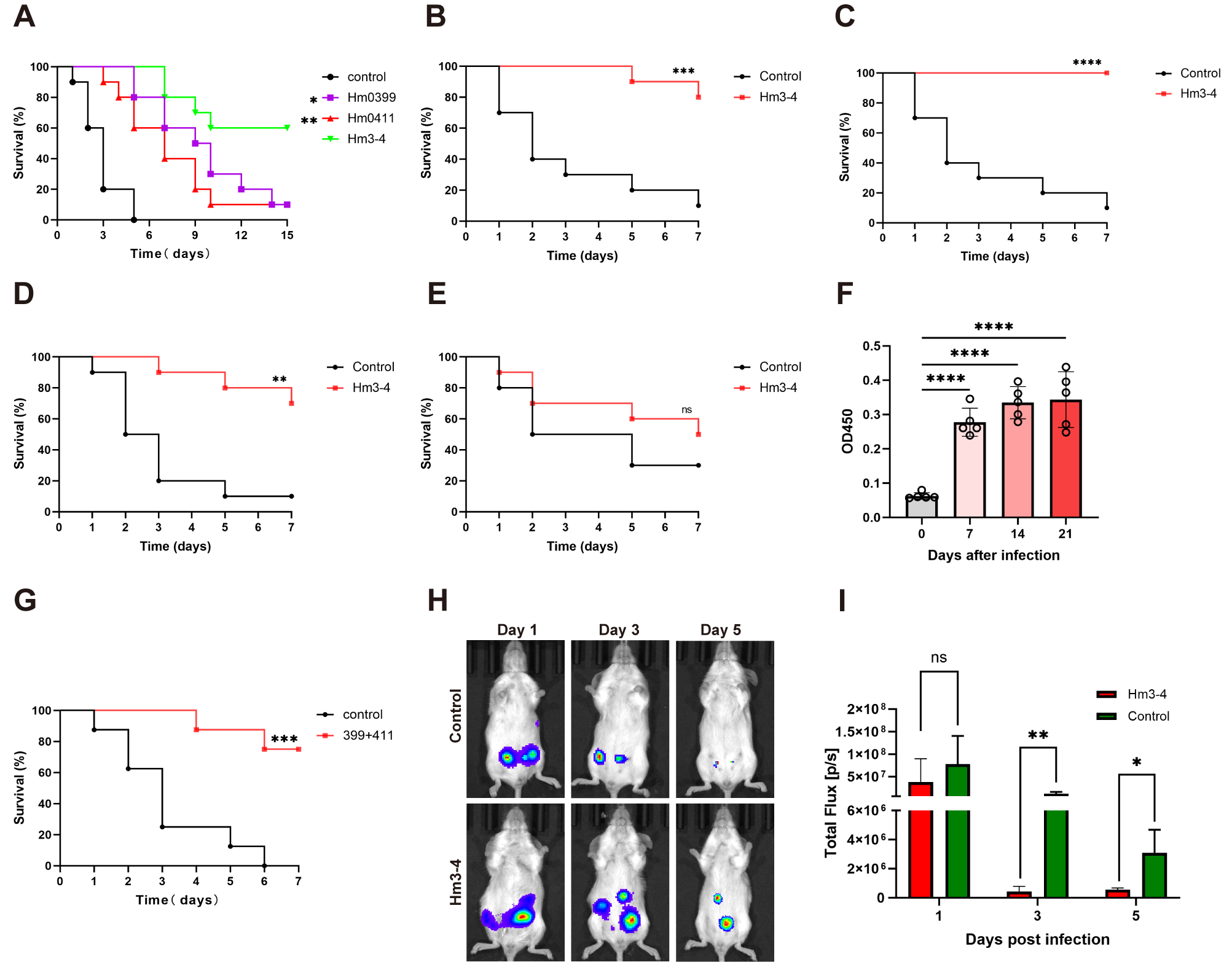


**Figure S5. The *in-vivo* protective efficacy of Hm3-4.** A, The protective efficacy when extending the observation period to 15 days. B to E, The protective efficacy on different *S. aureus* strains including Newman (**B**), NCTC 8325-4 (**C**), ST59 (**D**), and DU1090 (**E**). *n* = 10 mice per group. Data are presented as mean ± SD and analyzed using GraphPad Prism software (v.10.1.2). *P* values were determined using simple survival analysis (Kaplan-Meier) in A, B, C, D, E, and G. *P* values were determined using ordinary one-way ANOVA with Dunnett's multiple comparison in F. *P* values were determined using unpaired *t*-test in I. ns, not significant; **P* < 0.05; ***P* < 0.01; ****P* < 0.001; *****P* < 0.0001.

**Table S1 Gene Sequences of Hm0399 and Hm0411**

**Hm0399 heavy chain gene sequence**

ATGGAGACAGACACACTCCTGCTATGGGTACTGCTGCTCTGGGTTCCAGGTTCCACTGGTGACGAGGTGCAGCTGGTGGAGTCTGGGGGAGGCTTGGTACAGCCGGGGGGGTCCCTGAGACTCTCCTGTGCAGCCTCTGGATTCACCTTTACCAAATTTGCCATGAACTGGGTCCGCCAGGCTCCAGGGAAGGGGCTGGAGTGGGTCTCAGCCATTAGTGGGAGTGGTGGACTTACATACTACGCACACTCCGTGAAGGGCCGGTTCTCCATCTCCAGAGACAATTCCAAGAACACTCTGTATCTGCAAATGAACAGCCTGAGAGCCGAGGACACGGCCGTATATTACTGTGCGAGACCCGCCGGGGGAAAAGAGCACGGTGACTATATAATTGACTGCTGGGGCCAGGGCACCCTGGTCACCGTCTCCTCAGCCTCCACCAAGGGCCCATCGGTCTTCCCCCTGGCACCCTCCTCCAAGAGCACCTCTGGGGGCACAGCGGCCCTGGGCTGCCTGGTCAAGGACTACTTCCCCGAACCGGTGACGGTGTCGTGGAACTCAGGCGCCCTGACCAGCGGCGTGCACACCTTCCCGGCTGTCCTACAGTCCTCAGGACTCTACTCCCTCAGCAGCGTGGTGACCGTGCCCTCCAGCAGCTTGGGTACCCAGACCTACATCTGCAACGTGAATCACAAGCCCAGCAACACCAAGGTGGACAAGAGAGTTGAGCCCAAATCTTGTGACAAAACTCACACATGCCCACCGTGCCCAGCACCTGAACTCCTGGGGGGACCGTCAGTCTTCCTCTTCCCCCCAAAACCCAAGGACACCCTCATGATCTCCCGGACCCCTGAGGTCACATGCGTGGTGGTGGACGTGAGCCACGAAGACCCTGAGGTCAAGTTCAACTGGTACGTGGACGGCGTGGAGGTGCATAATGCCAAGACAAAGCCGCGGGAGGAGCAGTACAACAGCACGTACCGTGTGGTCAGCGTCCTCACCGTCCTGCACCAGGACTGGCTGAATGGCAAGGAGTACAAGTGCAAGGTCTCCAACAAAGCCCTCCCAGCCCCCATCGAGAAAACCATCTCCAAAGCCAAAGGGCAGCCCCGAGAACCACAGGTGTACACCCTGCCCCCATCCCGGGAGGAGATGACCAAGAACCAGGTCAGCCTGACCTGCCTGGTCAAAGGCTTCTATCCCAGCGACATCGCCGTGGAGTGGGAGAGCAATGGGCAGCCGGAGAACAACTACAAGACCACGCCTCCCGTGCTGGACTCCGACGGCTCCTTCTTCCTCTATAGCAAGCTCACCGTGGACAAGAGCAGGTGGCAGCAGGGGAACGTCTTCTCATGCTCCGTGATGCATGAGGCTCTGCACAACCACTACACGCAGAAGAGCCTCTCCCTGTCCCCGGGTAAA

**Hm0399 light chain gene sequence**

ATGGAGACAGACACACTCCTGCTATGGGTACTGCTGCTCTGGGTTCCAGGTTCCACTGGTGACCAGTCTGTGTTGACTCAGCCACCCTCAGCGTCTGGGACCCCCGGGCAGAGGGCCACCATCTCCTGTTCTGGAAGCAGTTCCAACATCGGAGGTAGCACTGTAATCTGGTACCAGCAGCTCCCAGGAACGGCCCCCAAACTCCTCATCTATAGTAATAATCAGCGGCCCTCAGGGGTCCCTGACCGATTCTCTGTCTCCAAGTCTGGCACCTCAGCCTCCCTGGCCATCAGTGGGCTCCAGTCTGAGGATGAGGCTAATTATTACTGTGCAGCATGGGATGACAGCCTGAAAGGTTGGGTGTTCGGCGGAGGGACCAAACTGACCGTCCTAGGTCAGCCCAAGGCTGCCCCCTCGGTCACTCTGTTCCCGCCCTCCTCTGAGGAGCTTCAAGCCAACAAGGCCACACTGGTGTGTCTCATAAGTGACTTCTACCCGGGAGCCGTGACGGTGGCCTGGAAGGCAGATAGCAGCCCCGTCAAGGCGGGAGTGGAGACCACCACACCCTCCAAACAAAGCAACAACAAGTACGCGGCCAGCAGCTATCTGAGCCTGACGCCTGAGCAGTGGAAGTCCCACAGAAGCTACAGCTGCCAGGTCACGCATGAAGGGAGCACCGTGGAGAAGACAGTGGCCCCTACAGAATGTTCA

**Hm0411 heavy chain gene sequence**

GGATCCGCCACCATGGACGCCATGAAAAGAGGCCTGTGCTGCGTGCTCCTGCTGTGCGGCGCTGTGTTCGTGAGCCCCGAGGTGCAGCTGGTGGAAAGCGGCCCCGAAGTGAAAAAGCCCGGAGCTTCTGTGAAGGTGTCTTGTAAAGCCACCGGCCACAACATTCTGACCTACGGCGTGAGCTGGGTCAGACAGGCCCCTGGCCAGGGACTGGAGTGGATGGGCTGGATCTCCGTTGACAAGGGCAATACCAACTACGCCCACGACTTCCAGGGCAGAGTGACCCTGATCACCGAGACAAGCACAAACACCGTGTACATGGAACTGCGGAGCCTGAGATCCGATGATACCGCTATCTACTACTGCGTGCGGGGCGGCGAGACATACCTGATGGACTACTGGGGCCAAGGCACCCTGGTGGCCGTCTCTAGCGCCTCTACAAAGGGACCTAGCGTCTTTCCTCTGGCCCCTAGCTCTAAGAGCACCAGCGGAGGAACCGCCGCCCTGGGTTGTCTGGTGAAGGATTACTTCCCAGAGCCCGTGACAGTGTCCTGGAACAGCGGCGCCCTCACCAGCGGCGTGCACACCTTTCCCGCCGTGCTGCAGAGCAGCGGCCTGTACTCTCTGTCTAGCGTGGTCACCGTGCCTAGCAGTAGCCTGGGCACCCAGACCTACATCTGCAACGTGAACCACAAGCCTTCTAATACCAAGGTGGACAAGAAAGTGGAACCTAAATCCTGCGACAAAACCCACACCTGCCCTCCATGTCCTGCCCCTGAACTGCTGGGCGGGCCTAGCGTGTTTCTGTTCCCCCCCAAGCCCAAGGACACCCTGATGATCAGCAGAACCCCTGAGGTGACCTGCGTGGTGGTGGACGTGTCCCACGAAGATCCTGAGGTGAAATTCAACTGGTACGTGGATGGCGTTGAGGTGCATAATGCCAAAACCAAGCCTAGAGAGGAACAGTACAACAGCACATATAGAGTTGTGTCAGTGCTGACAGTTCTGCACCAGGACTGGCTGAACGGCAAGGAATACAAGTGCAAGGTGTCTAACAAGGCCCTGCCTGCTCCAATCGAGAAGACAATCAGCAAGGCCAAGGGACAGCCTCGGGAACCCCAGGTGTATACACTGCCTCCTAGCCGGGACGAGCTGACAAAGAACCAGGTGAGCCTGACTTGTCTGGTCAAGGGCTTCTACCCCAGCGATATCGCCGTCGAGTGGGAGAGCAACGGACAACCTGAGAATAACTACAAGACAACCCCTCCAGTGCTGGACAGCGACGGCTCCTTCTTCCTGTACAGCAAGCTGACCGTGGACAAGAGCAGGTGGCAGCAGGGCAACGTGTTCAGCTGCAGCGTGATGCACGAGGCCCTGCACAACCACTACACACAGAAGTCCCTGAGCCTGAGCCCTGGCAAGTGACTCGAG

**Hm0411 light chain gene sequence**

GGATCCGCCACCATGGACGCCATGAAAAGAGGCCTGTGTTGCGTGCTGCTGCTGTGCGGCGCCGTCTTTGTGTCCCCTCAGCCCGTGCTGACCCAGCCTCCATCCGTGTCCGGCACACCTGGACAAAGAGTGACAATGAGCTGCTCCGGCACCACAAGCAACATCGGCGGAAATACCGTGAACTGGTACAGGCAGCTGCCTGGCGCTGCTCCTACCCTGCTCATCTACACCACAAAGTACCGGCCTAGCGGCGTGCCTGATAGAATCAGCGGCTCTAAGAGCGGCACCAGCGCCAGCCTGGCCATCTCTGAACTGCAAAGCGAAGATGAGGCCGACTACTACTGCGCCGCTTGGGACGACAGCCTGCGGGGCTGGGTGTTCGGCGGCGGAACCAAGGTGACCGTGCTTGGACAGCCAAAGGCCGCCCCTAGCGTGACCCTGTTCCCCCCCTCTAGCGAGGAACTGCAGGCCAACAAGGCCACCCTGGTGTGCCTGATCAGCGACTTCTACCCCGGCGCCGTGACCGTGGCCTGGAAAGCCGATAGCTCTCCTGTGAAGGCAGGCGTTGAAACCACAACCCCTAGCAAGCAGAGCAACAACAAATACGCCGCTTCTTCTTATCTGAGCCTGACACCCGAGCAGTGGAAGTCCCACAGAAGCTACAGCTGTCAGGTGACCCACGAGGGCAGCACAGTCGAGAAGACCGTGGCCCCTACAGAGTGCAGCTGACTCGAG

**Table S2 |** Intermolecular donor-acceptor distances for hydrogen bonds

|  | **Hla** | **0411 Fab Heavy chain** | | **0411 Fab Light chain** | |
| --- | --- | --- | --- | --- | --- |
|  | **Residues** | **Residues** | **Length(Å)** | **Residues** | **Length(Å)** |
| **Hydrogen bonds** | D44(OD1) |  |  | Y54(OH) | 3.43 |
|  | D44(OD2) |  |  | Y54(OH) | 2.39 |
|  | D235(OD2) | Y32(OH) | 3.48 |  |  |
|  | K240(NZ) |  |  | N32(OD1) | 2.80 |
| **Salt bridges** | D44(OD2) |  |  | K53(NZ) |  |
|  | K237(NZ) | D106(OD2) | 3.43 |  |  |
|  | K240(NZ) |  |  | D94(OD1) | 3.10 |
|  | K240(NZ) |  |  | D94(OD2) | 3.46 |
|  | E287(OE1) |  |  | K67(NZ) | 3.86 |

|  | **Hla** | **0399 Fab Heavy chain** | | **0399 Fab Light chain** | |
| --- | --- | --- | --- | --- | --- |
|  | **Residues** | **Residues** | **Length(Å)** | **Residues** | **Length(Å)** |
| **Hydrogen bond**s | D44(OD2) |  |  | Q54(NE2) | 3.46 |
|  | K46(NZ) |  |  | Q54(OE1) | 3.87 |
|  | R236(NE) |  |  | Q54(OE1) | 3.63 |
|  | K237(NZ) |  |  | Y50(OH) | 2.59 |
| **Salt bridges** | E287(OE1) |  |  | K67(NZ) | 3.79 |

**Table S3 |** **Statistics for Cryo-EM data collection, refinement, and validation**

|  | Hla in complex with Fab 411 |
| --- | --- |
| **Data collection** |  |
| Voltage (kV) | 300 |
| Microscope | FEI Titan Arctica |
| Camera | K2 (Gatan) |
| Magnification (calibrated) | 75000X |
| Electron exposure (e^–^/Å^2^) | 60 |
| Exposure rate (e^–^/Å^2^/s) | 16.02 |
| Number of frames collected per micrograph | 32 |
| Automation software | SerialEM |
| Defocus range (μm) | –1.2 to –1.8 |
| Pixel size (Å) | 1.04 |
| **Overall map processing** |  |
| Micrographs used | 4,401 |
| Symmetry imposed | C7 |
| Initial particle images | 1,544,750 |
| Final particle images | 52,889 |
| Resolution at 0.143 FSC of masked reconstruction (Å) | 3.42 |
| Map sharpening B factor (Å^2^) | -78.3 |
| **Local map refinement** |  |
| Refinement package | Phenix v1.19 |
| Model composition |  |
| Non-hydrogen atoms | 32,213 |
| Protein residues | 3,871 |
| R.m.s. deviations |  |
| Bond lengths (Å) | 0.004 |
| Bond angles (°) | 1.111 |
| *B* factors (Å^2^) |  |
| Protein | 465.77 |
| Validation |  |
| MolProbity score | 1.92 |
| Clashscore | 8.28 |
| Poor rotamers (%) | 0 |
| Ramachandran plot |  |
| Favored (%) | 92.48 |
| Allowed (%) | 7.41 |
| Disallowed (%) | 0 |
| Cb outliers (%) | 0 |
| CaBLAM outliers (%) | 5.53 |
